# Increased CAPG inhibits ferroptosis to drive tumor proliferation and sorafenib resistance in hepatocellular carcinoma via the WDR74-p53-SLC7A11 pathway

**DOI:** 10.7150/ijbs.111419

**Published:** 2025-08-22

**Authors:** Bing Quan, Fan Yao, Wenfeng Liu, Bei Tang, Miao Li, Shenxin Lu, Jinghuan Li, Rongxin Chen, Zhenggang Ren, Xin Yin

**Affiliations:** 1 Liver Cancer Institute, Zhongshan Hospital, Fudan University, Shanghai 200032, China.; 2 National Clinical Research Center for Interventional Medicine, Shanghai 200032, China.

**Keywords:** CAPG, ferroptosis, tumor proliferation, sorafenib resistance, hepatocellular carcinoma

## Abstract

Hepatocellular carcinoma (HCC) presents a global therapeutic challenge owing to its aggressive tumor progression and limited treatment options. Therefore, identifying novel therapeutic targets is urgently needed. In this study, we identified CAPG as a top candidate gene that is upregulated in HCC tissues and predicts poor clinical prognosis, based on proteomic sequencing, public database analysis, and immunohistochemistry. The biological role of CAPG in HCC tumorigenesis was investigated using cell lines, xenograft models, and pulmonary metastasis models. We found that CAPG depletion inhibited tumor proliferation and metastasis both in vivo and in vitro. Functional assays were also performed to assess the effects of CAPG on sorafenib-induced ferroptosis. Colony formation assays, IC_50_ assays, qPCR, and Western blot analyses were conducted to examine the relationship between CAPG expression and sorafenib treatment. Notably, CAPG was upregulated following sorafenib exposure and contributed to sorafenib resistance. RNA sequencing, ChIP sequencing, co-immunoprecipitation, and ubiquitination assays were further employed to elucidate the molecular mechanisms involving CAPG. Mechanistically, CAPG promoted gene expression by inducing WDR74 transcription, which modulated the interaction between p53 and MDM2, resulting in p53 degradation. Our findings demonstrate that CAPG drives tumor proliferation and sorafenib resistance by inhibiting ferroptosis, suggesting that CAPG may serve as a promising target in HCC.

## Introduction

Hepatocellular carcinoma (HCC) is the third leading cause of cancer-related death worldwide and is characterized by high recurrence and metastasis rates 1. In the early stages of HCC, surgical resection, liver transplantation 2, and local ablation 3 are considered effective curative therapies. Nonetheless, up to 70% of patients experience recurrence within 5 years after resection or local ablation 4. However, owing to the lack of specific clinical symptoms, most patients are diagnosed at a mid-to-late stage, often missing the optimal window for radical treatment 5. Transcatheter arterial chemoembolization (TACE) 6, hepatic artery infusion chemotherapy (HAIC) 7 and other locoregional therapies remain essential options for managing advanced HCC. In addition, targeted therapies, including sorafenib, regorafenib, lenvatinib 8-10, as well as immunotherapy 11, 12, have shown promising treatment outcomes. Despite these advances, effective management of advanced-stage HCC remains a challenge. Therefore, novel therapeutic strategies for HCC are urgently needed.

Ferroptosis, a novel form of iron-dependent cell death that is driven by lipid peroxidation, was first described in 2012 13. It is distinct from traditional forms of programmed cell death, such as apoptosis, autophagy, pyroptosis, and necrosis. Hallmark features of ferroptosis include intracellular redox imbalance and iron overload, leading to decreased levels of glutathione peroxidase 4 (GPX4) and subsequent cell death. Ferroptosis plays a significant role in tumor progression 14. Moreover, evidence suggests that inhibition of solute carrier family 7 member 11 (SLC7A11) by sorafenib leads to glutathione (GSH) depletion and GPX4 inactivation, ultimately inducing ferroptosis 15, 16. Thus, suppression of ferroptosis is a critical mechanism underlying sorafenib resistance 17. Taken together, these findings suggest that pharmacological modulation of ferroptosis may offer substantial clinical benefits in HCC treatment.

Macrophage-capping protein (CAPG, also known as MCP) is a member of the gelsolin family, which regulates actin assembly 18. CAPG is distributed in both the cytoplasm and nucleus 19. Interestingly, nuclear CAPG has a stronger effect on promoting invasion than cytoplasmic CAPG 20. The CAPG gene shares homologous sequences with genes encoding basic helix-loop-helix (bHLH) DNA-binding proteins and therefore has the potential to modulate gene transcription 21. A previous study confirmed that CAPG enhances breast cancer metastasis by promoting transcription of the gene stanniocalcin 1 (STC1) 22. CAPG upregulation has been implicated in the proliferation, invasion, and metastasis of multiple tumors, including prostate, breast, pancreatic, and gastric cancers, as well as HCC 20, 23-27. However, the functional role of CAPG in HCC has only been demonstrated *in vitro*. Moreover, sequencing data have suggested that CAPG may act as a negative regulator of ferroptosis in HCC 28. Nevertheless, the molecular mechanism through which CAPG regulates malignant phenotypes and ferroptosis of HCC remains unclear.

In this study, we identified elevated CAPG expression in HCC and demonstrated that high CAPG levels are associated with poor clinical prognosis. Suppression of CAPG expression inhibited tumor growth, metastasis, and sorafenib resistance both *in vitro* and *in vivo*. Mechanistically, CAPG protected HCC cells from ferroptosis-induced cell death by promoting the expression of WD repeat-containing protein 74 (WDR74). WDR74, in turn, disrupted the interaction between tumor protein p53 (TP53) and mouse double minute 2 homolog (MDM2), thereby upregulating SLC7A11 protein levels. Our findings suggest that CAPG functions as a ferroptosis suppressor in HCC and may represent a novel therapeutic target for treatment.

## Methods

### Tissue specimens

In this study, five fresh HCC tissues and paired adjacent normal tissues were collected from Zhongshan Hospital, affiliated with Fudan University, for proteomic sequencing. The study was approved by the Ethics Committee of Zhongshan Hospital, and informed consent was obtained from all participants (KY2024441). Additionally, we retrospectively analyzed tumor tissues from 68 independent HCC patients at Zhongshan Hospital using immunohistochemistry (IHC). A tissue microarray containing 77 paired HCC and adjacent normal tissues was obtained from Puxu Company. All patients received sorafenib following liver resection. Based on overall survival (OS), the 77 patients were categorized into a sorafenib-sensitive group and a sorafenib-resistant group. The clinical characteristics of both groups are provided in **Table S1.**

### Tandem mass tag (TMT)-labeled quantitative proteomics

To identify differences in protein expression between the HCC tissues and paired adjacent normal tissues, protein extraction and digestion were conducted using the filter-aided sample preparation method described by Matthias Mann 29. According to the manufacturer's instructions (Thermo Scientific, USA), 100 μg of peptide mixture from each sample was labeled with TMT reagents. All samples were analyzed by Shanghai Applied Protein Technology. Differentially expressed proteins (DEPs) were defined by a fold change > 1.6 or < 0.625 and a p-value < 0.05.

### Cell lines and cell culture

The normal liver cell line L02 and the human HCC cell lines Hep3B, HepG2, and PLC/PRF/5 were obtained from the Cell Bank of the Chinese Academy of Sciences. Huh7 cells were acquired from the Japanese Cancer Research Resources Bank, while MHCC97H and HCCLM3 cells were established at the Liver Cancer Institute of Fudan University. All cells were cultured in Dulbecco's Modified Eagle Medium (DMEM; Gibco) supplemented with 10% fetal bovine serum (FBS; Gibco) and 1% penicillin‒streptomycin (Invitrogen, USA) and maintained at 37 °C in a humidified incubator with 5% CO_2_.

To establish sorafenib-resistant (SR) cells, 97H and Huh7 cells were continuously exposed to sorafenib (HY-10201) for approximately 4 months, with concentrations gradually increased from 0.5 μM to a maximum tolerated dose of 10 μM. The resulting 97H-SR and Huh7-SR cell lines were subsequently cultured in medium containing 1 μM sorafenib to preserve acquired resistance.

### Cell transfection

CAPG-targeting small hairpin RNA (shRNA) lentivirus, CAPG overexpression lentivirus, small interfering RNA (siRNA) targeting WDR74, and the WDR74 overexpression plasmid were purchased from Genomeditech (Shanghai, China). Stable CAPG-knockdown and CAPG-overexpressing cell lines were generated by lentiviral infection, followed by selection with 10 μg/mL puromycin for two cycles of 48 hours each. For transient knockdown or overexpression of WDR74, cells were transfected with siRNAs and plasmids using Lipofectamine 3000 transfection reagent (Thermo Fisher Scientific, USA). The sequences of the shRNAs and siRNAs used in this study are listed in **Table S2**.

### Quantitative real-time polymerase chain reaction (qRT‒PCR)

Total RNA was extracted using the RNA-Quick Purification Kit (EZBioscience, China) following the manufacturer's instructions. Complementary DNA (cDNA) was synthesized using 4× EZscript Reverse Transcription Mix II (EZBioscience), and single-stranded cDNA was amplified using 2× SYBR Green qPCR Master Mix (ROX2 plus; EZBioscience). Relative mRNA expression levels were calculated using the (2- ΔΔCt) method. The sequences of the primers used in this study are listed in **Table S3**.

### Western blotting analysis and antibodies

Total protein was extracted from cells using RIPA lysis buffer (Beyotime, China) supplemented with 1 mM phenylmethylsulfonyl fluoride (PMSF) and 2 mM phosphatase inhibitor (both from Beyotime, China). Protein concentrations were measured using a BCA kit (Beyotime, China). Proteins were separated by SDS‒PAGE and transferred to PVDF membranes (Millipore). Membranes were blocked with 5% skim milk and incubated with primary antibodies overnight at 4 °C, followed by incubation with secondary antibodies for 1 hour at room temperature. Protein bands were visualized using ECL reagents (Yeasen, China) and detected with an imaging system.

The antibodies that were used in this study included antibodies against CAPG (ab155688, Abcam, UK), SLC7A11 (ab307601, Abcam, UK), ACSL4 (acyl-CoA synthetase long-chain family member 4, ab155282, Abcam, UK), TFRC (transferrin receptor protein 1, ab269513, Abcam, UK), MDM2 (ab259265, Abcam, UK), p53 (ab26, Abcam, UK), WDR74 (20631-1-AP, Proteintech, USA), Nrf2 (nuclear factor erythroid 2-related factor 2, 80593-1-RR, Proteintech, USA), GAPDH (glyceraldehyde-3-phosphate dehydrogenase, AF1186, Beyotime, China), HA (AF0039, Beyotime, China), and HIF-1α (hypoxia inducible factor 1 alpha, abs154588, Absin, China).

### IHC staining and hematoxylin and eosin (H&E) staining

Five-micron-thick paraffin-embedded sections of clinical tissues, mouse subcutaneous tumors, and mouse metastatic tissues were subjected to IHC staining. After dewaxing and dehydration, the sections were incubated with antibodies against CAPG (ab155688, Abcam, UK), SLC7A11 (ab307601, Abcam, UK), Ki-67 (ab15580, Abcam, UK), and 4-hydroxynonenal (4-HNE, ab48506, Abcam, UK). Sections were then incubated with secondary antibodies and subsequently scanned using a NanoZoomer S60 slide scanner (Hamamatsu Photonics). H&E staining was performed using a H&E staining kit (Beyotime, China) according to the manufacturer's protocol.

### Cell viability assay

HCC cells (2,000 cells per well) were seeded in 96-well plates and treated with varying concentrations of test reagents. Cell viability was assessed using a CCK-8 kit (Yeasen, China) according to the manufacturer's instructions. After 2 hours of incubation at 37 °C, absorbance (Ab) was measured at 450 nm using a microplate spectrophotometer. Cell viability was calculated using the following formula: Cell viability (%) = (Ab of experimental group - Ab of blank group) / (Ab of control group - Ab of blank group) × 100.

### Colony formation assay

Cells were seeded in six-well plates at a density of 1,000 cells per well and cultured for 2 weeks. At the end of the incubation period, cells were fixed with 4% paraformaldehyde and stained with crystal violet solution (Beyotime, China). Images were captured, and colony numbers were quantified using ImageJ software.

### EdU assay

The EdU incorporation assay was performed using the BeyoClick™ EdU-555 Kit (Beyotime, China). Cells were incubated with 10 μM EdU for 2 hours at 37 °C and then fixed with 4% formaldehyde for 15 minutes at room temperature. Cells were permeabilized with 0.3% Triton X-100 for 15 minutes, followed by incubation with the EdU reaction cocktail in the dark for 30 minutes. Nuclei were counterstained with Hoechst 33342 for 10 minutes in the dark. Fluorescence images were acquired using a fluorescence microscope.

### Wound-healing assay

Cells were seeded into six-well plates for the wound-healing assay. When cells reached approximately 90% confluence, a scratch was created using a sterile 200 μL pipette tip. The monolayer was then washed and incubated in low-serum medium. Images were obtained at 0 and 48 hours after wounding and analyzed using ImageJ software.

### Transwell migration assay and Matrigel invasion assay

For migration and invasion assays, 2,000 cells were seeded into the upper chambers of 12-well Transwell plates in 150 μL of serum-free medium. For the invasion assay, the upper chambers were pre-coated with Matrigel (Corning, USA). The lower chambers were filled with 750 μL of DMEM supplemented with 20% FBS. After 48 hours of incubation at 37 °C, cells on the lower surface of the membrane were fixed with 4% paraformaldehyde and stained with crystal violet solution (Beyotime, China). Images were captured using an inverted microscope and analyzed with ImageJ software.

### Half-maximal inhibitory concentration (IC50) assay

Cells were seeded in 96-well plates at a density of 2,000 cells per well. After a 24-hour incubation, various concentrations of sorafenib were added, and cells were incubated for an additional 48 hours. The culture medium was then removed and replaced with 100 μL of fresh medium containing 10% CCK-8 reagent (Yeasen, China). Following a 2-hour incubation at 37 °C, absorbance was measured at 450 nm using a Multiskan Spectrum microplate spectrophotometer (Thermo Scientific, USA).

### Immunofluorescence assay

Cells were fixed with 4% paraformaldehyde for 15 minutes, permeabilized with 0.1% Triton X-100 for 10 minutes, and blocked with 10% goat serum for 1 hour. Cells were then incubated with primary antibodies overnight at 4 °C, followed by incubation with fluorescently labeled secondary antibodies at room temperature for 1 hour. Nuclei were counterstained with DAPI for 15 minutes. Fluorescent images were acquired under a confocal microscope.

### Transmission electron microscopy (TEM)

Cells were harvested and fixed in 2.5% glutaraldehyde at 4 °C for 2.5 hours, followed by three washes in phosphate-buffered saline (PBS) and post-fixation in 1% osmium tetroxide (OsO_4_) at 4 °C for 2 hours. After dehydration through a graded ethanol series, samples were embedded in Spurr's resin. Ultrathin sections (70 nm) were cut and stained with uranyl acetate and lead citrate before examination with a transmission electron microscope.

### Lipid peroxidation detection

Cells were stained with C11-BODIPY 581/591 (Invitrogen, USA) for 30 minutes at 37 °C and then washed with PBS. After trypsinization, cells were collected and resuspended in 150 μL of PBS. Lipid peroxidation levels were assessed by flow cytometry using a 488 nm laser to detect fluorescence intensity. An equal number of single cells (10,000) were analyzed per sample. Data were processed using FlowJo software.

### Malondialdehyde (MDA) detection

MDA levels in HCC cells and mouse tumor tissues were measured using the MDA Assay Kit (S0131, Beyotime, China). Briefly, cells were lysed with 0.1 mL of lysis buffer per 1 × 10⁶ cells, followed by centrifugation at 10,000 × *g* for 10 minutes to collect the supernatant. Next, 100 μL of cell lysate was transferred to 96-well plates, mixed with 200 μL of MDA detection working solution, and the absorbance at 532 nm was measured using a multifunctional microplate reader.

### GSH detection

GSH levels were determined using the GSH and GSSG Assay Kit (S0053, Beyotime, China). Processed samples (10 μL) were mixed with 150 μL of total glutathione detection working solution and incubated at room temperature for 5 minutes. Then, 50 μL of 0.5 mg/mL NADPH solution was added and thoroughly mixed. After a 25-minute reaction, absorbance at 412 nm was measured using a multifunctional microplate reader.

### Intracellular ferrous iron measurement

Cells were seeded in 6-cm plates and incubated overnight to allow adherence. After treatment with sorafenib (10 μM) for 24 hours, cells were collected and resuspended. Intracellular ferrous iron levels were then measured using the Cell Ferrous Iron Colorimetric Assay Kit (E-BC-K881-M, Elabscience, China). Specifically, approximately 1 × 10⁶ cells were lysed in 0.2 mL of lysis buffer. Then, 80 μL of cell lysate was transferred to a 96-well plate, followed by the addition of the ferrous ion detection working solution. After a 10-minute incubation at 37 °C, absorbance at 593 nm was measured using a multifunctional microplate reader.

### RNA sequencing (RNA-seq) analysis

Total RNA was extracted from Vector PLC and CAPG-shPLC cells, as well as Control PLC and CAPG OE PLC cells. Following RNA quality assessment and library construction, libraries were sequenced on an Illumina NovaSeq 6000 platform by Beijing Novogene Co., Ltd. (Beijing, China). Differentially expressed genes (DEGs) were identified with the following criteria: |log_2_(fold change)| > 1 and p-value < 0.05.

### Chromatin immunoprecipitation-sequencing (ChIP-seq) and ChIP‒qPCR

Approximately 2 × 10^7^ LM3 cells were fixed in 1% formaldehyde for 10 minutes at room temperature. Crosslinking was quenched by the addition of 125 mM glycine, followed by a 5-minute incubation. Cells were rinsed three times with PBS, scraped, and collected into centrifuge tubes. ChIP-seq analysis was performed by LC-Bio Technologies Co., Ltd. (Hangzhou, China).

For ChIP‒qPCR, 1 × 10⁷ LM3 and PLC cells were crosslinked with 1% formaldehyde, lysed, and sonicated to shear chromatin into fragments. Chromatin complexes were immunoprecipitated overnight at 4 °C using either anti-CAPG antibody (ab155688, Abcam, UK) or control rabbit IgG. Target DNA enrichment was quantified using qPCR.

### Dual-luciferase reporter assay

Human embryonic kidney 293T (HEK-293T) cells were seeded in 24-well plates and transfected once they reached approximately 70% confluency. The following plasmid combinations were used: (1) promoter control plasmid + transcription factor control plasmid, (2) promoter control plasmid + CAPG plasmid, (3) WDR74 plasmid + transcription factor control plasmid, and (4) WDR74 plasmid + CAPG plasmid. After 48 hours, firefly luciferase (pGL3) and Renilla luciferase activities were measured using the Dual-Luciferase Reporter Assay System (Promega), following the manufacturer's instructions. Promoter activity was calculated as the ratio of firefly luciferase to Renilla luciferase.

### Coimmunoprecipitation (co-IP) Assay

Co-IP was performed using the Co-IP Kit (P2179S, Beyotime, China) according to the manufacturer's protocol. Briefly, protein A + G magnetic beads were incubated with 10 μg/mL of antibody at 4 °C for 1 hour. Cells were lysed in RIPA buffer and incubated with antibody-coated beads overnight at 4 °C. Beads were washed with PBS and eluted with elution buffer. Immunoprecipitated complexes were analyzed using western blotting.

### Ubiquitylation assay

Target cells were transfected with expression vectors encoding HA-tagged ubiquitin. Twenty-four hours post-transfection, cells were treated with 20 μM MG132 for 4 hours, followed by washing and lysis. Ubiquitinated p53 was immunoprecipitated using an anti-p53 antibody and analyzed using western blotting with an anti-HA antibody.

### Xenograft mouse model

Five-week-old male BALB/c nude mice were purchased from the Laboratory Animal Center of Fudan University (Shanghai, China). All mice were housed under specific pathogen-free conditions. A total of 5 × 10^6^ cells were subcutaneously injected into the axilla to establish the subcutaneous xenograft model. Tumor length (L) and width (W) were measured every 3 days for approximately 4 weeks, and tumor volume was calculated using the formula: (L × W^2^)/2. At the end of the experiment, the mice were sacrificed, and the tumors were harvested for ferroptosis assessment and IHC analysis. For sorafenib treatment, once tumors reached approximately 200 mm^3^ in volume, mice were administered sorafenib (30 mg/kg) or saline (vehicle control) by oral gavage daily.

### In vivo lung cancer metastasis model

To establish the lung metastasis model, mice were injected via the tail vein with 3 × 10^6^ HCCLM3 cells transfected with either control or CAPG-targeting shRNA. After 5 weeks, all mice were sacrificed, and lung tissues were harvested. H&E staining was performed, and the number of metastatic lung nodules was quantified.

### Bioinformatic analysis

HCC mRNA expression data were obtained from The Cancer Genome Atlas (TCGA) (https://www.cancer.gov/about-nci/organization/ccg/research/structural-genomics/tcga) and the Gene Expression Omnibus (GEO) database (https://www.ncbi.nlm.nih.gov/geo/). The GEPIA2 (http://gepia2.cancer-pku.cn) platform was used to analyze the potential correlation between CAPG and WDR74 expression.

### Statistical analysis

All statistical analyses were performed using SPSS 26.0 and GraphPad Prism 9.0. Data are presented as mean ± standard deviation (SD). Comparisons between two groups were performed using Student's t-test, and comparisons among multiple groups were analyzed using one-way ANOVA. Survival analysis was conducted using the Kaplan‒Meier method with the log-rank test. A p-value < 0.05 was considered statistically significant. *P values less than 0.05, **P values less than 0.01, ***P values less than 0.001.

## Results

### CAPG is upregulated in HCC tissues and correlates with poor prognosis

**Figure 1:**
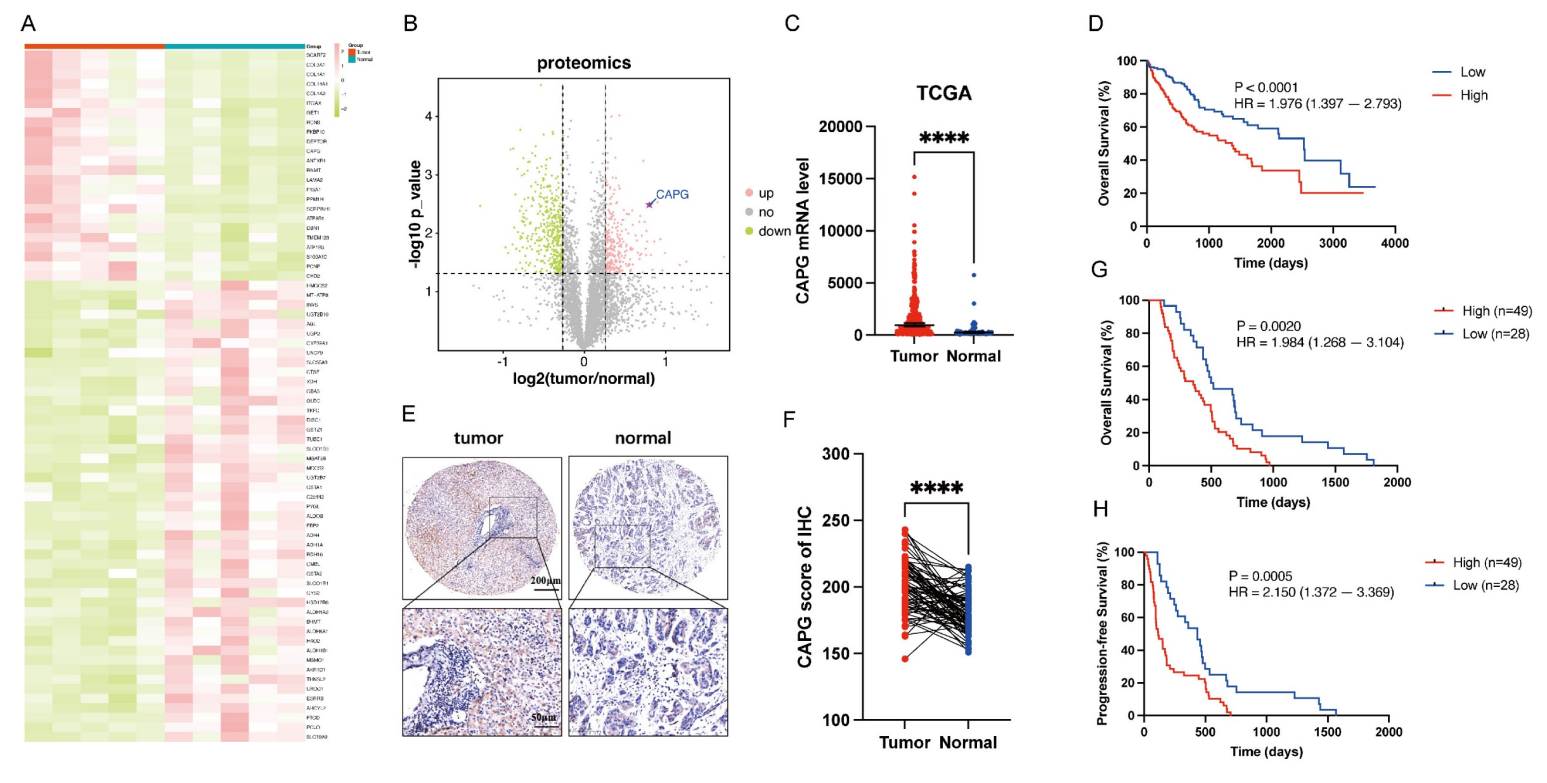
** CAPG is upregulated in HCC tissues and associated with poor prognosis. A.** Heatmap of differentially expressed proteins (DEPs) in paired primary HCC and adjacent normal tissues (pink: upregulated; green: downregulated). **B.** Volcano plot of DEPs (pink: upregulated; green: downregulated; grey: not significant). **C.** CAPG expression is significantly higher in HCC tissues than in peritumoral tissues based on TCGA data. **D.** Kaplan-Meier plots analysis showing overall survival (OS) in HCC samples stratified based on CAPG expression using TCGA data. **E.** Representative immunohistochemistry (IHC) images of CAPG in HCC tissue microarrays. **F.** Statistical analysis of CAPG expression scores in paired HCC and adjacent normal tissues. **G, H.** Kaplan-Meier plots showing the association between CAPG expression and overall survival (OS) or recurrence-free survival (RFS) in HCC patients. *P < 0.05, **P < 0.01, ***P < 0.001, ns = not significant.

To identify DEPs associated with HCC, we performed proteomics analysis on five paired primary HCC tissues and adjacent normal tissues. The resulting heatmap and volcano plot revealed that CAPG was among the most highly upregulated DEPs in HCC tissues compared with adjacent normal tissues (**Fig. 1A, B**). The complete dataset is presented in **Table S4**. To validate these findings, CAPG expression was assessed in HCC and peritumor tissues using TCGA database (**Fig. 1C**) and three publicly available GEO datasets (GSE54236, GSE14520, and GSE121248) (**Fig. S1A-C**). These datasets consistently showed increased CAPG expression in HCC tissues. In the TCGA cohort, higher CAPG expression was also associated with shorter overall survival (**Fig. 1D**). Additionally, we examined CAPG expression in 77 paired HCC and adjacent non-tumor tissues using tissue microarrays analysis, confirming its overexpression in tumor samples (**Fig. 1E, F**). Kaplan‒Meier survival analysis further revealed that patients with elevated CAPG levels had significantly poorer OS and progression-free survival (PFS) (**Fig. 1G, H**).

Next, we measured CAPG expression across several HCC cell lines using qPCR and Western blotting. CAPG expression was relatively higher in 97H and LM3 cells and lower in Huh7 and PLC cells (**Fig. S1D, E**). Based on these results, 97H and LM3 cells were selected for stable knockdown experiments, while Huh7 and PLC cells were used to generate stable overexpression lines for subsequent functional analyses.

### CAPG knockdown inhibits HCC cell proliferation and metastasis *in vitro* and *in vivo*

**Figure 2:**
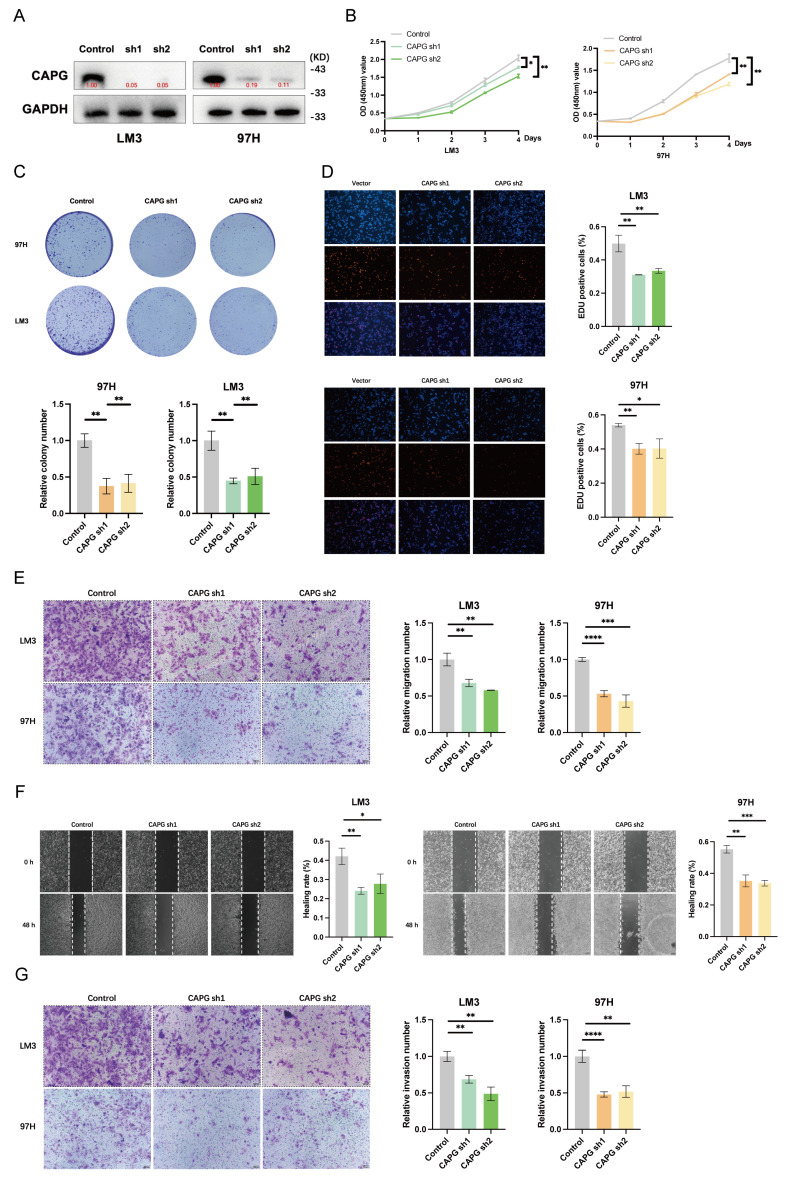
** CAPG promotes HCC cell proliferation, migration, and invasion. A.** Validation of CAPG knockdown efficiency in HCC cells. **B.** CCK-8 assays showing reduced proliferation in CAPG-silenced HCC cells.** C.** Colony formation assays in HCC cells with CAPG knockdown. **D.** EdU assays in HCC cells after CAPG knockdown. **E.** Transwell migration assays demonstrating decreased migration following CAPG silencing. **F.** Wound healing assays showing impaired migration in CAPG-silenced HCC cells. **G.** Matrigel invasion assays assessing invasive ability after CAPG knockdown. *P < 0.05, **P < 0.01, ***P < 0.001, ns = not significant.

To investigate the role of CAPG in HCC cell proliferation, migration, and invasion, we first confirmed the efficiency of CAPG knockdown and overexpression by assessing CAPG protein levels (**Fig. 2A, S2A**). CCK-8 proliferation assays, colony formation assays, and EdU incorporation assays demonstrated that CAPG silencing significantly reduced the viability of 97H and LM3 cells. In contrast, CAPG overexpression in Huh7 and PLC cells led to increased cell viability (**Fig. 2B-D, S2B-D**). Consistently, Transwell migration assays, wound healing assays, and Matrigel invasion assays revealed that CAPG knockdown markedly impaired the migratory and invasive capacities of 97H and LM3 cells, whereas CAPG overexpression enhanced these malignant phenotypes (**Fig. 2E-G, S2E-G**). These results suggest that CAPG promotes HCC cell proliferation, migration, and invasion *in vitro*.

**Figure 3:**
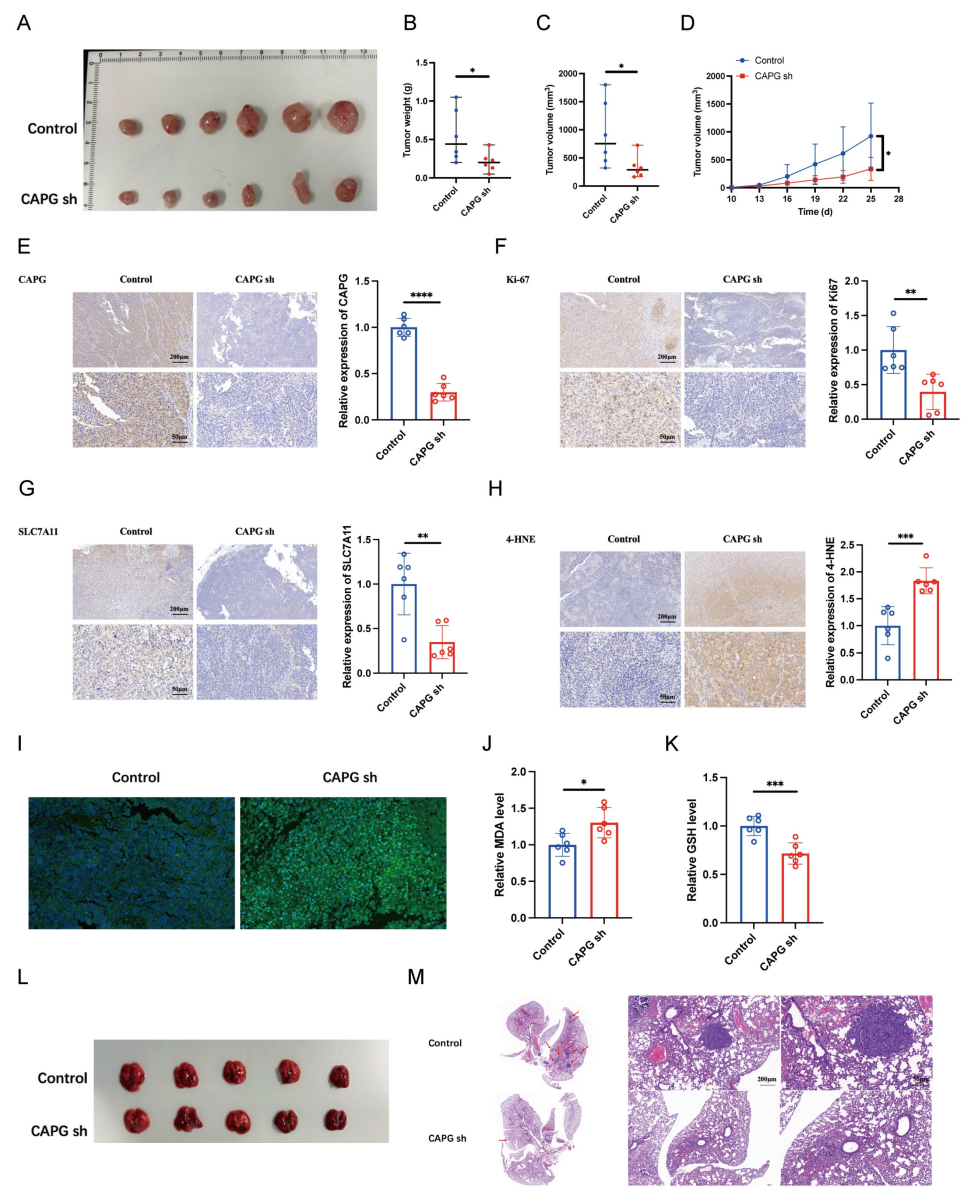
** CAPG silencing suppresses HCC tumor proliferation and metastasis by inducing ferroptosis *in vivo*. A.** Representative images of tumors harvested from BALB/c nude mice subcutaneously injected with 5 × 10^6^ LM3 NC or shCAPG cells. **B.** Tumor weight in each group (n = 6). **C.** Tumor volumes in each group (n = 6). **D.** Tumor growth curves (n = 6). **E-H.** Representative IHC images for (CAPG, Ki67, SLC7A11, and 4-HNE in tumor tissues. **I.** Representative immunofluorescence (IF) images of tumors stained with the C11-BODIPY probe. **J.** Relative malondialdehyde (MDA) levels in each group (n = 6). **K.** Relative glutathione (GSH) levels in each group (n = 6). **L.** Representative images of lung tissues showing metastatic nodules (n = 5). **M.** Representative H&E-stained sections of metastatic lung nodules from both groups. *P < 0.05, **P < 0.01, ***P < 0.001, ns = not significant.

To further assess the oncogenic role of CAPG *in vivo*, LM3 cells transduced with either control shRNA (shNC) or CAPG-targeting shRNA (shCAPG) were subcutaneously injected into BALB/c nude mice. CAPG knockdown significantly suppressed tumor weight and volume (**Fig. 3A-D**). IHC analysis revealed decreased Ki67 expression in tumors derived from shCAPG cells (**Fig. 3E, F**).

To evaluate the effect of CAPG on metastasis *in vivo*, LM3 shNC or shCAPG cells were injected intravenously into BALB/c nude mice. After 5 weeks, the mice were sacrificed, and the lung tissues were collected. Histological analysis showed a markedly reduced number of metastatic lung nodules in the shCAPG group compared to controls (**Fig. 3L, M**). Collectively, these findings indicate that CAPG plays a critical role in promoting HCC proliferation and metastasis both *in vitro* and *in vivo*.

### CAPG overexpression suppresses sorafenib-induced ferroptosis in HCC cells

Given that CAPG mRNA and protein expression are significantly altered by erastin 28, we investigated whether CAPG regulates ferroptosis to promote HCC progression. To assess ferroptosis levels in tumor tissues, we performed IHC staining for SLC7A11 and 4-HNE, and measured lipid peroxidation, MDA levels, and GSH content. Notably, shCAPG tumors exhibited reduced expression of SLC7A11 and 4-HNE (**Fig. 3G, H**), increased lipid peroxidation (**Fig. 3I**) and MDA levels (**Fig. 3J**), and decreased GSH levels (**Fig. 3K**), indicating enhanced ferroptosis.

Sorafenib can act as a ferroptosis inducer in HCC 30, 31. Here, we successfully established SR 97H and Huh7 cell lines and validated their resistance using IC_50_ assays, CCK-8 assays, qPCR, and western blotting (**Fig. S3A-D**). We further confirmed that sorafenib induces ferroptosis in both parental and SR HCC cells, as evidenced by elevated MDA levels and lipid peroxidation (**Fig. S4A, B**). Based on these findings, we next explored the potential relationship between CAPG expression and sorafenib-induced ferroptosis.

**Figure 4:**
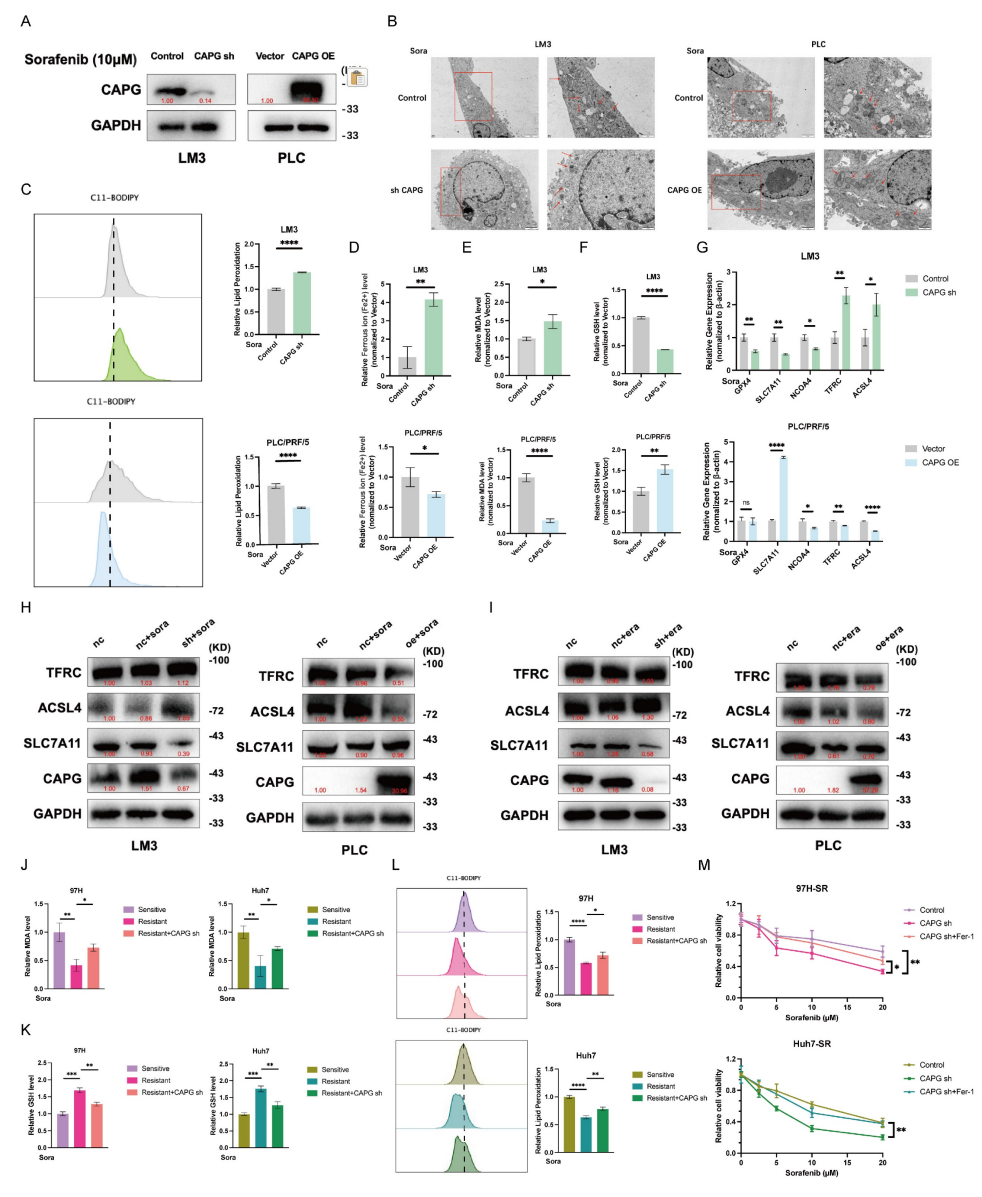
** CAPG regulates sorafenib-induced ferroptosis in HCC cells. A.** Western blot validation of CAPG knockdown (sh) and overexpression (OE) in HCC cells. **B.** Representative transmission electron microscopy (TEM) images of mitochondrial morphology in CAPG-sh and CAPG-OE cells treated with 10 μM sorafenib for 24 hours. Red arrows indicate mitochondria. **C-F.** Relative levels of lipid reactive oxygen species (ROS) (**C**), intracellular ferrous iron (Fe^2+^) (**D**), MDA (**E**), and GSH (**F**) in CAPG-sh and CAPG-OE cells after 24 hours of 10 μM sorafenib treatment. **G.** Relative mRNA expression of ferroptosis-related genes in CAPG-sh and CAPG-OE cells after 24 hours of sorafenib treatment. **H, I.** Relative protein levels of ferroptosis-related genes in CAPG-sh and CAPG-OE cells after 24 h of treatment with 10 μM sorafenib **(H)** or 10 μM erastin **(I)**. **J-L.** Relative levels of MDA (**J**), GSH (**K**), and lipid ROS (**L**) in CAPG-knockdown, sorafenib-resistant HCC cells treated with 10 μM sorafenib.** M.** Relative viability of CAPG-knockdown, sorafenib-resistant HCC cells treated with various concentrations of sorafenib, with or without 1 μM ferrostatin-1 (Fer-1), for 48 h.

We first confirmed the efficacy of CAPG knockdown and overexpression in HCC cells following 24-hour incubation with sorafenib (10 μM) (**Fig. 4A, S5A**). Mitochondrial morphological changes, such as reduced volume, loss of cristae, and increased membrane density, are well-established features of ferroptosis 13. TEM revealed that CAPG knockdown intensified these mitochondrial abnormalities and enhanced sorafenib-induced cell death, while CAPG overexpression attenuated these effects (**Fig. 4B**). We then evaluated additional ferroptosis-related parameters, including lipid peroxidation, intracellular iron levels, MDA levels, and GSH levels. CAPG knockdown led to elevated lipid peroxidation, iron, and MDA levels, along with reduced GSH levels. In contrast, CAPG overexpression had opposing effects (**Fig. 4C-F, S5B-E**). We further assessed the mRNA and protein expression of ferroptosis-associated genes following sorafenib treatment (10 μM, 24 h). CAPG knockdown reduced the expression of the ferroptosis-suppressor gene SLC7A11, while increasing the expression of ferroptosis-promoting genes TFRC and ACSL4. Conversely, CAPG overexpression reversed these effects (**Fig. 4G, H, S5F, G**).

Similar findings were observed following treatment with erastin (10 μM), a known ferroptosis inducer and *SLC7A11* inhibitor, which shares the same molecular targets as sorafenib (**Fig. 4I, S5H**). Next, we explored whether CAPG knockdown enhances ferroptosis sensitivity in SR HCC cells. As expected, SR cells were less responsive to erastin than sorafenib-sensitive cells. However, CAPG knockdown restored erastin sensitivity in SR cells (**Fig. S6A, B**). Furthermore, CAPG silencing in resistant cells led to increased lipid peroxidation and MDA levels, along with reduced GSH levels, upon sorafenib treatment (**Fig. 4J-L**).

CAPG knockdown also enhanced the sensitivity of SR cells to sorafenib, as evidenced by decreased cell viability. This sensitizing effect was reversed by co-treatment with *ferrostatin-1* (*Fer-1*), a ferroptosis inhibitor (**Fig. 4M**). To further confirm the involvement of ferroptosis, we compared the effects of several cell death inhibitors. Only Fer-1, but not other inhibitors such as *TTM* (cuproptosis inhibitor), *Z-VAD-FMK* (apoptosis inhibitor), *necrosulfonamide* (necroptosis inhibitor), *N-Acetylcysteine* (ROS inhibitor), or chloroquine (autophagy inhibitor), mitigated sorafenib-induced cell death **Fig. S6C**). These findings indicate that the increase in CAPG knockdown-induced cell death following sorafenib treatment is mediated by ferroptosis.

### CAPG promotes sorafenib resistance in HCC cells and is upregulated by sorafenib treatment

**Figure 5:**
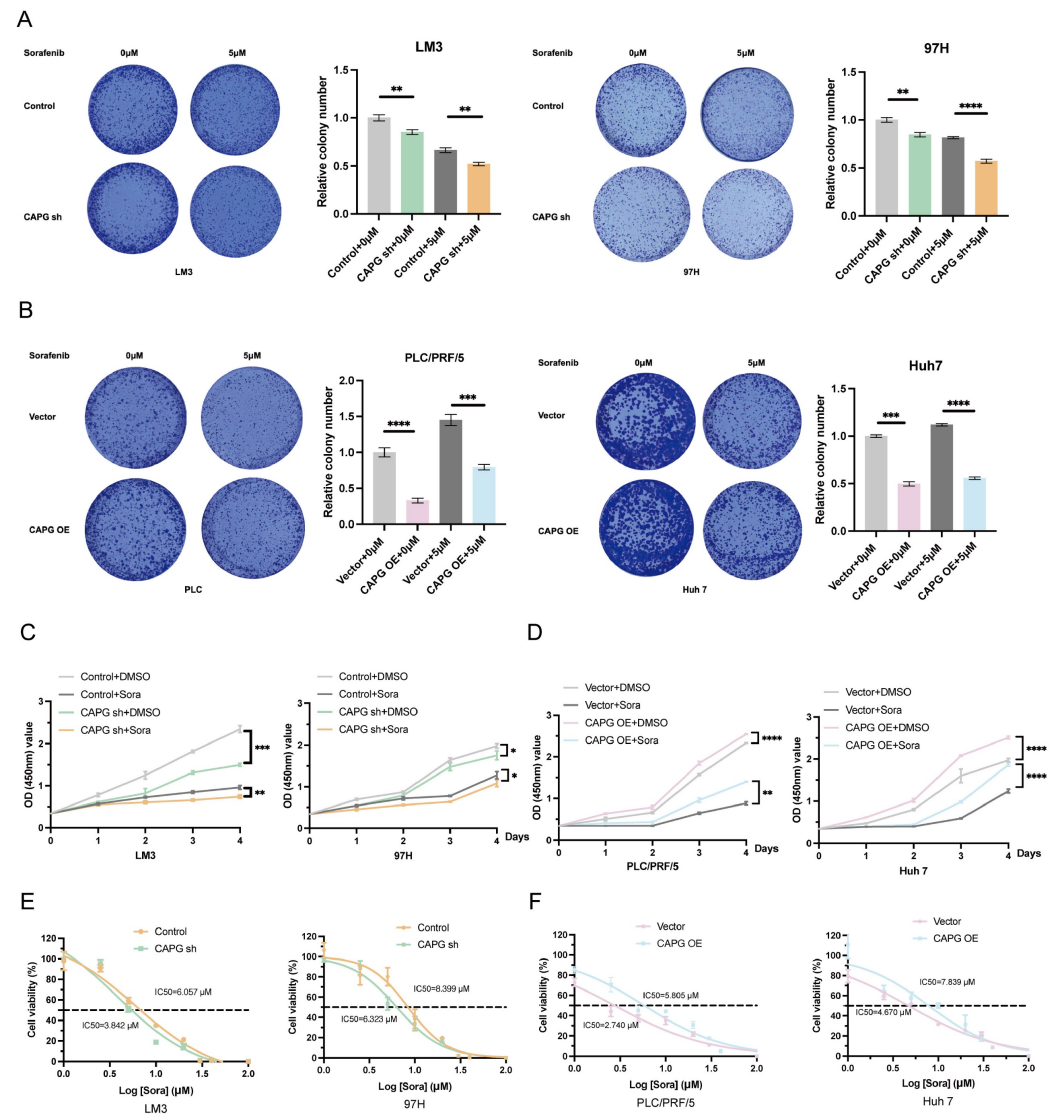
** CAPG promotes sorafenib resistance in HCC cells. A, B.** Colony formation assays of CAPG knockdown (sh) and overexpression (OE) cells treated with or without sorafenib (5 μM) for 24 hours. **C, D.** Relative absorbance (OD) values of CAPG-sh and CAPG-OE cells treated with or without 10 μM sorafenib for 24 hours. **E, F.** Half-maximal inhibitory concentration IC_50_ of CAPG sh and CAPG-OE cells after treatment with various concentrations of sorafenib for 24 hours. *P < 0.05, **P < 0.01, ***P < 0.001, ns = not significant.

Next, we explored the relationship between CAPG and resistance to sorafenib. CCK-8 and colony formation assays showed that CAPG knockdown significantly increased sorafenib-induced cell death, while CAPG overexpression conferred resistance (**Fig. 5A-D**). The IC_50_ values of sorafenib were lower in CAPG-silenced HCC cells compared with controls (LM3: 3.842 μM vs. 6.057 μM; 97H: 6.323 μM vs. 8.399 μM;** Fig. 5E**), whereas IC_50_ values were higher in CAPG-overexpressing cells (PLC: 5.805 μM vs. 2.740 μM; Huh7: 7.839 μM vs. 4.670 μM;** Fig. 5F**). Consistent findings were observed in SR HCC cell lines (**Fig. S7A-C**). These results suggest that CAPG deficiency enhances sorafenib sensitivity in HCC cells.

**Figure 6:**
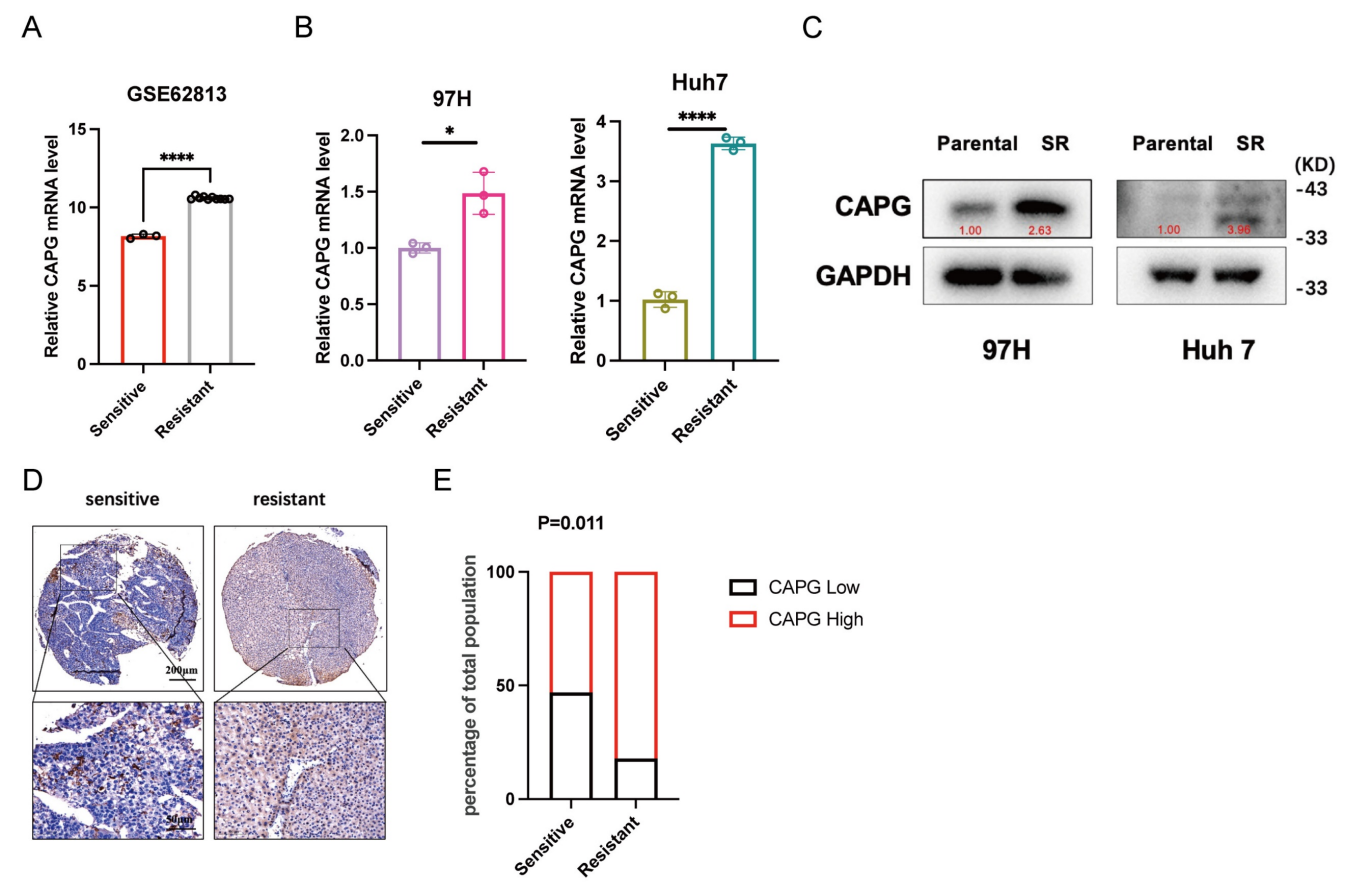
** Sorafenib treatment upregulates CAPG expression and nuclear translocation in HCC cells. A.** CAPG mRNA expression level increased in sorafenib-resistant HepG2 cells based on the GSE62813 dataset. **B, C.** CAPG mRNA and protein expression levels are elevated in sorafenib-resistant HCC cells. **D.** Representative tissue microarray images showing CAPG expression. **E.** Negative correlation between CAPG expression levels and sorafenib sensitivity. *P < 0.05, **P < 0.01, ***P < 0.001, ns = not significant.

We then assessed whether sorafenib regulates CAPG expression. Analysis of the GSE62813 dataset revealed significantly increased CAPG mRNA levels in HepG2-SR cells compared with parental cells (**Fig. 6A**). This upregulation was further confirmed in SR cell lines using qPCR and western blotting (**Fig. 6B, C**). Tissue microarrays analysis also revealed a negative correlation between CAPG expression and sorafenib sensitivity (**Fig. 6D, E**). Finally, we examined the temporal dynamics of CAPG expression following sorafenib treatment. HCC cells treated with 10 μM sorafenib exhibited a time-dependent increase in CAPG expression, peaking at approximately 24 hours. However, CAPG levels declined after 48 hours, possibly due to prolonged drug exposure inducing translational repression (**Fig. S8**).

### CAPG activates the transcription of WDR74 in HCC cells

**Figure 7:**
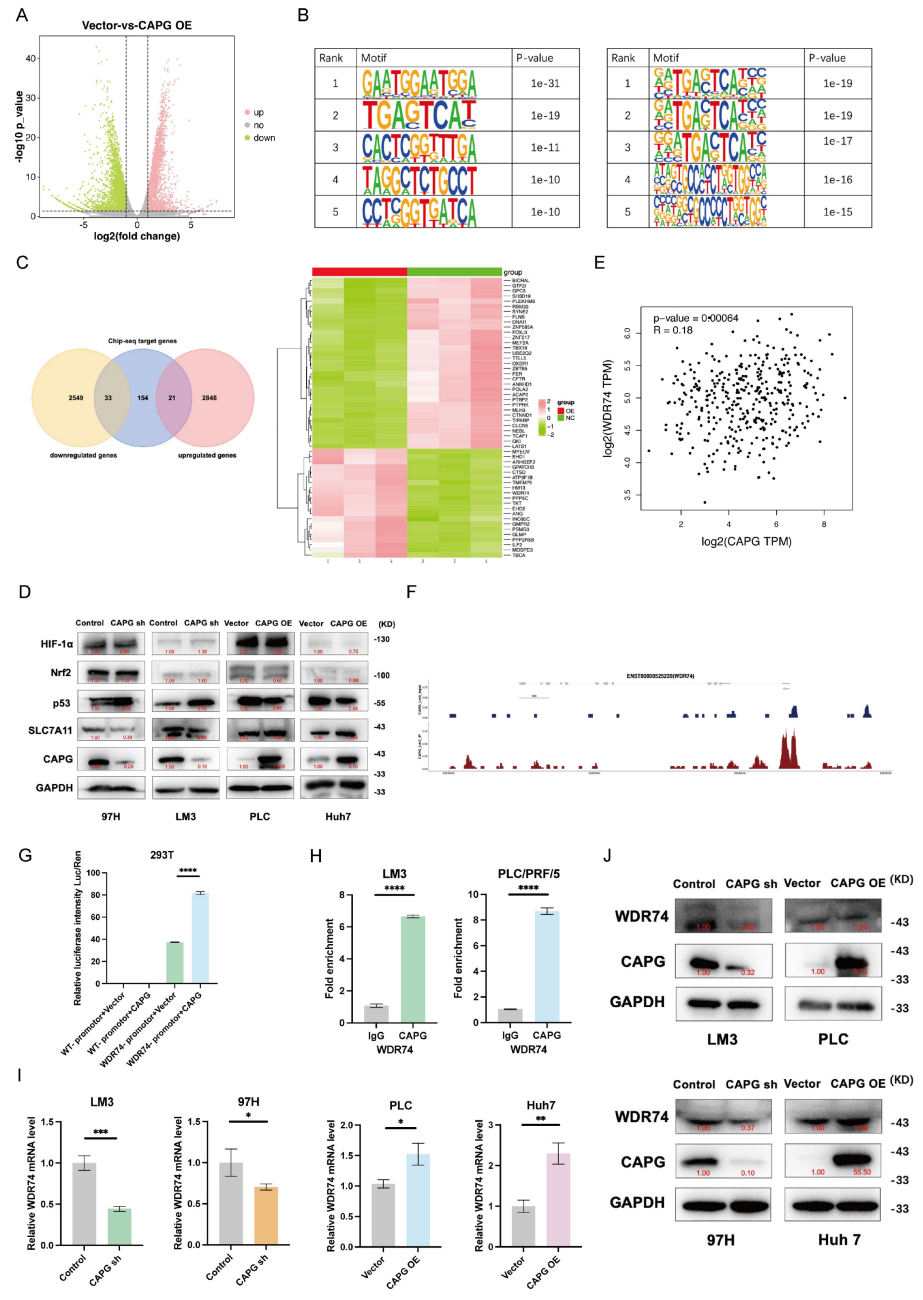
** CAPG is involved in the activation of WDR74 transcription in HCC cells. A.** Volcano plot with differentially expressed genes in CAPG overexpression group compared with control group in PLC cells. **B.** Top five predicted CAPG-binding de novo motifs (left) and known motifs (right) with the most significant *P*-values control identified in LM3 cells. **C.** Venn diagram -and heatmap display overlapping genes identified by RNA-seq and ChIP-seq analyses. **D.** WDR74 knockdown or overexpression affects p53 expression but not HIF-1α and Nrf2 expression. **E.** Correlation analysis of CAPG and WDR74 expression levels based on TCGA data. **F.** ChIP-seq peaks indicating CAPG binding at the WDR74 promoter region. **G.** Dual-luciferase reporter assay assessing transcriptional activation in 293T cells. **H.** ChIP-qPCR confirmation of CAPG binding to the WDR74 promoter in 97H and Huh7 cells. **I, J.** WDR74 mRNA and protein expression levels in CAPG-sh and CAPG-OE HCC cells. *P < 0.05, **P < 0.01, ***P < 0.001, ns = not significant.

To further investigate the mechanism underlying CAPG function in HCC, we performed RNA-seq and ChIP-seq to identify CAPG-regulating genes. RNA-seq analysis revealed 2,869 upregulated and 2,582 downregulated genes in CAPG-overexpressing PLC cells compared with control cells (**Fig. 7A**). ChIP-seq identified 208 genes with peaks located at promoter transcription start sites. The top five predicted de novo and known CAPG-binding motifs were identified (**Fig. 7B**). Through intersection analysis of the RNA-seq and ChIP-seq datasets, we identified 54 candidate transcription targets directly regulated by CAPG (**Fig. 7C**). Western blotting revealed that CAPG affected the expression of SLC7A11 via p53, rather than through Nrf2 or HIF-1α (**Fig. 7D**). Sorafenib treatment upregulates p53 expression. Thus, we confirmed that the observed interaction between WDR74 and p53 was not attributable to sorafenib-mediated p53 induction (**Fig. S9**).

Based on these findings, we hypothesized that WDR74, a gene previously reported to promote p53 degradation in melanoma, may be a major downstream effector of CAPG 32. In support of this, TCGA data demonstrated a significant positive correlation between CAPG and WDR74 expression in HCC (**Fig. 7E**). ChIP-seq revealed CAPG-binding peaks within the promoter region of WDR74 (chromosome 11, position 62,841,380-62,841,911) **(Fig. 7F)**. This binding was further validated by dual-luciferase reporter assays and ChIP‒qPCR (**Fig. 7G, H**). Moreover, qPCR and western blot analyses demonstrated that WDR74 expression was reduced following CAPG knockdown and increased upon CAPG overexpression (**Fig. 7I, J**). These results indicate that CAPG transcriptionally activates WDR74 by directing binding to its promoter in HCC cells.

### WDR74 regulates protein binding between p53 and MDM2

**Figure 8:**
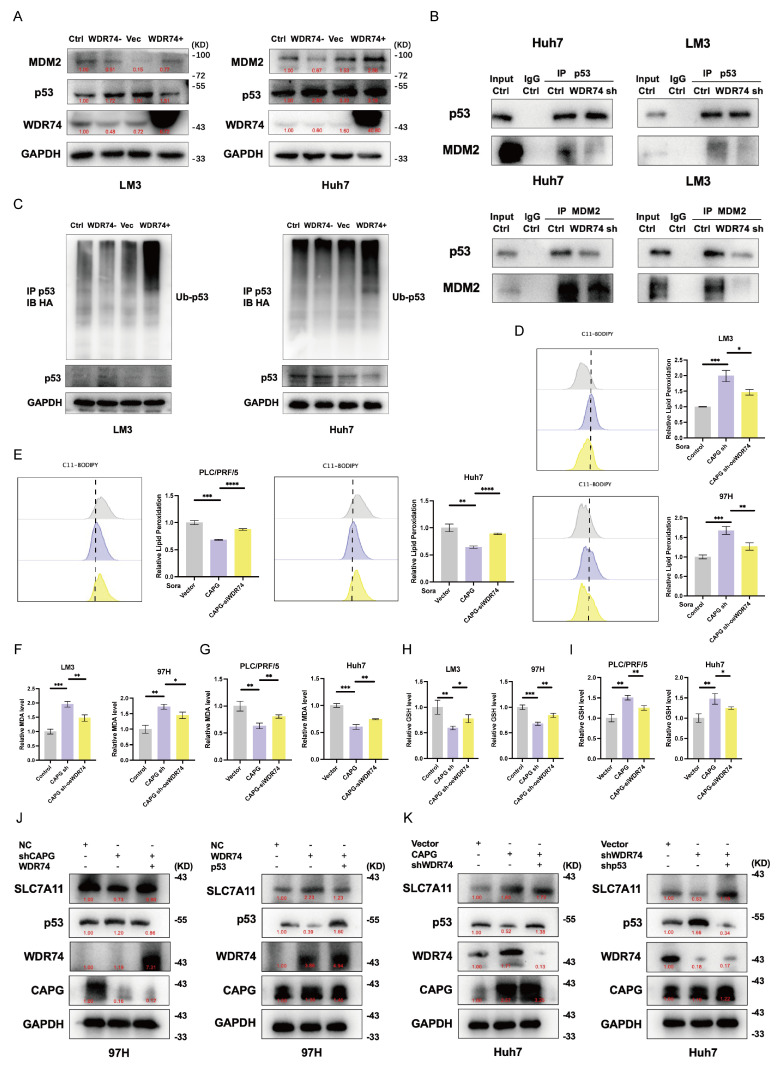
**WDR74 knockdown disrupts the interaction between p53 and MDM2. A.** Western blot analysis of p53 and MDM2 protein expression in WDR74-knockdown and WDR74 overexpressing HCC cells. **B.** HCC cells were transfected with siWDR74 for 48 hours. Co-immunoprecipitation (Co-IP) analysis was performed to assess the interaction between p53 and MDM2. Cell lysates were immunoprecipitated using anti-p53 or anti-MDM2 antibodies, followed by immunoblotting with the indicated antibodies. **C.** WDR74 promotes p53 ubiquitination. Cells were transfected with Ub-HA plasmids and treated with 10 μM MG132 for 3 hours. p53 Immunoprecipitated using anti-p53 antibodies, and ubiquitinated p53 was detected by anti-HA immunoblotting. **D-I.** Relative lipid ROS levels (**D, E**), MDA levels (**F, G**), and GSH levels (**H, I**) in CAPG-knockdown cells with or without WDR74 overexpression and in CAPG-overexpressing cells with or without WDR74 knockdown. **J, K.** Western blot analysis of proteins involved in the WDR74-p53-SLC7A11 pathway in 97H and Huh7 cells. *P < 0.05, **P < 0.01, ***P < 0.001, ns = not significant.

To elucidate the mechanisms by which WDR74 interacts with p53 in HCC, we examined the role of WDR74 in the MDM2-p53 signaling axis. As shown in **Fig. 8A**, WDR74 knockdown increased p53 protein levels and decreased MDM2 protein levels. Conversely, WDR74 overexpression reduced p53 expression while elevating MDM2 levels. To further validate whether WDR74 modulates p53 degradation through the ubiquitin-proteasome pathway, we performed co-IP and p53 ubiquitination assays. The Co-IP results revealed that WDR74 knockdown impaired the interaction between p53 and MDM2 (**Fig. 8B**). Consistently, WDR74 knockdown led to reduced p53 ubiquitination. In contrast, WDR74 overexpression enhanced p53 ubiquitination and promoted its degradation (**Fig. 8C**).

Next, we explored whether WDR74 mediates the effects of CAPG on ferroptosis. Cotransfection with the WDR74 plasmid partially reversed the increase in lipid peroxidation, MDA levels, and the reduction in GSH levels (**Fig. 8D, F, H**). Conversely, WDR74 knockdown mitigated the effects of CAPG overexpression on these ferroptosis markers (**Fig. 8E, G, I**).

To further investigate the CAPG-WDR74-p53-SLC7A11 regulatory axis, we modulated the expression of CAPG, WDR74, and p53 in HCC cells via lentiviral transduction, siRNA transfection, and plasmid overexpression. CAPG knockdown significantly downregulated both WDR74 and SLC7A11 protein levels; however, these effects were reversed by WDR74 overexpression. Additionally, p53 overexpression attenuated the WDR74-induced increase in SLC7A11 expression (**Fig. 8J, K**).

Finally, RNA-seq analysis of WDR74-silenced PLC cells identified ferroptosis suppressor and ferroptosis driver genes under WDR74 control. Among them, SLC7A11 was a notable overlapping gene (**Fig. S10A**). Collectively, these results demonstrate that CAPG regulates the p53/SLC7A11 pathway via WDR74, contributing to ferroptosis suppression in HCC cells.

To further elucidate the role of WDR74 in the CAPG-induced malignant phenotype, rescue experiments were performed. Results from the CCK-8, EdU, Transwell migration, Matrigel invasion assays, and sorafenib IC_50_ analysis revealed that CAPG silencing inhibited the proliferation, migration, and invasion of LM3 cells. However, these inhibitory effects were reversed by WDR74 overexpression. Conversely, CAPG overexpression promoted the growth, mobility, and invasiveness of Huh7 cells, which were attenuated by WDR74 knockdown (**Fig. S10B-F**).

### CAPG/WDR74/p53/SLC7A11 axis regulates sorafenib-induced ferroptosis *in vivo*

**Figure 9:**
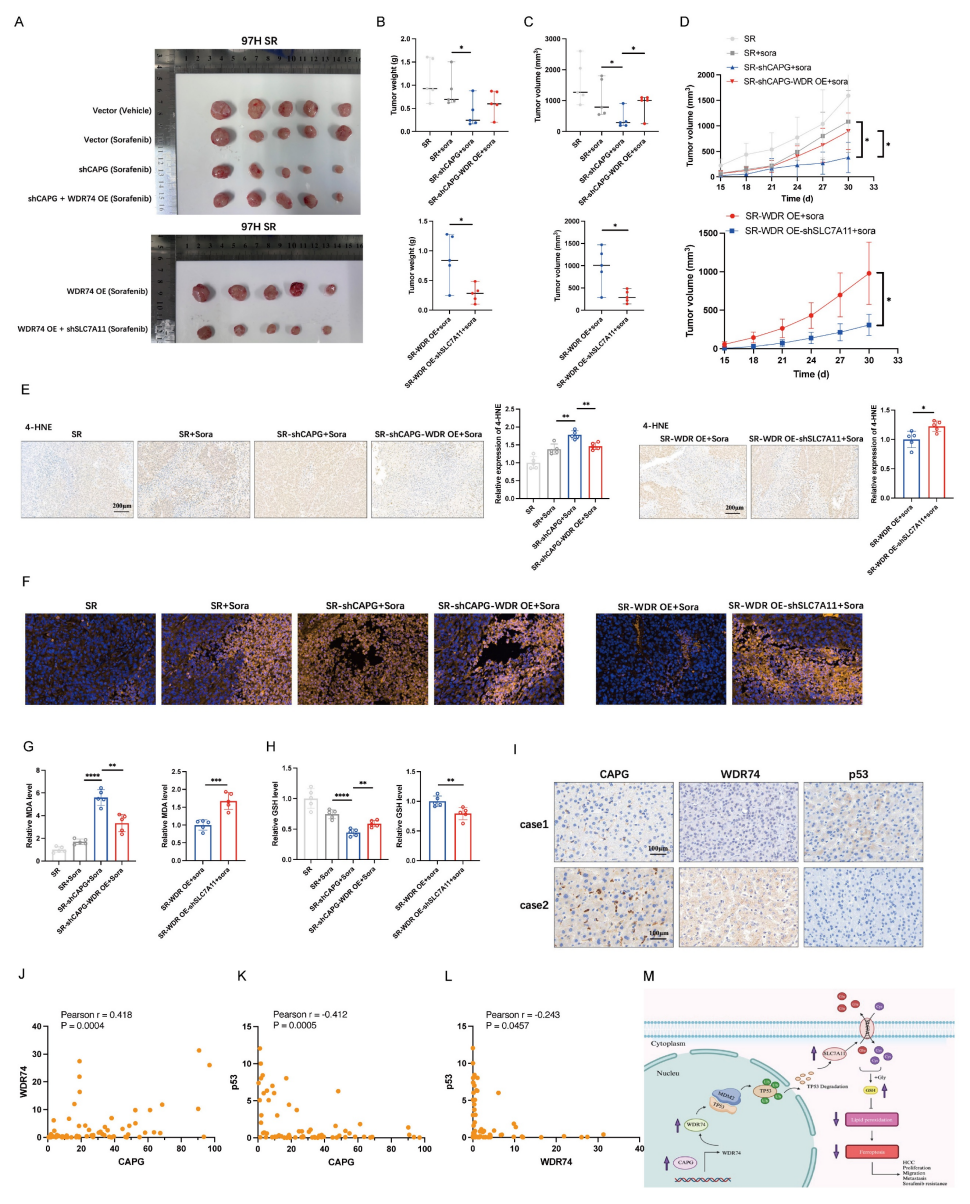
** CAPG/WDR74/p53/SLC7A11 axis regulates sorafenib-induced ferroptosis *in vivo*. A.** Representative tumor images from BALB/c nude mice injected with different HCC cell lines. **B.** Tumor weight in each group (n = 5). **C.** Tumor volume in each group (n = 5). **D.** Tumor growth curves (n = 5). **E.** Representative IHC images showing 4-HNE staining in tumor tissues. **F.** Representative immunofluorescence (IF) staining with the C11-BODIPY probe in tumor sections.** G.** Relative malondialdehyde (MDA) level in each group (n = 5). **H.** Relative glutathione (GSH) level in each group (n = 5). **I.** Representative IHC staining images of CAPG, WDR74, and p53 in HCC tissue were displayed. **J-L.** The correlation between CAPG and WDR74, CAPG and p53, and WDR74 and p53 was analyzed based on IHC score. **M.** Schematic diagram summarizing the proposed molecular mechanism. *P < 0.05, **P < 0.01, ***P < 0.001, ns = not significant.

To validate our findings *in vivo*, we established xenograft models using sorafenib-resistant 97H cells with stable CAPG knockdown, WDR74 overexpression, SLC7A11 knockdown, or control treatments. Sorafenib monotherapy only mildly inhibited tumor growth *in vivo*. CAPG knockdown significantly reduced the tumor weight, volume, and growth rate. This suppression was partially reversed by WDR74 overexpression, while SLC7A11 knockdown mitigated the tumor-promoting effects (**Fig. 9A-D**). As expected, ferroptosis-related markers, including 4-HNE staining, C11-Bodipy fluorescence intensity, MDA levels, and GSH levels, further confirmed the involvement of the CAPG/WDR74/p53/SLC7A11 axis in mediating sorafenib-induced ferroptosis *in vivo* (**Fig. 9E-H**). Additionally, we used CAPG-overexpressing Huh7 cells to establish xenograft models, which further validated the *in vivo* relevance of this regulatory axis (**Fig. S11A-I**).

To further confirm the expression relationships among CAPG, WDR74, and p53, IHC staining was performed on HCC tissue microarrays. Representative images are shown in **Fig. 9I**. Correlation analysis demonstrated that CAPG expression was positively correlated with WDR74 but negatively correlated with p53 expression. Similarly, WDR74 expression was inversely correlated with p53 levels (**Fig. 9J-L**).

## Discussion

Although the morbidity and mortality rates of patients with HCC have declined in recent years, the efficacy of currently available treatment strategies remains unsatisfactory 33. Therefore, further investigations into novel therapeutic targets and biomarkers are urgently needed to improve clinical outcomes for HCC patients. In this study, we demonstrated that CAPG promotes tumor proliferation and resistance to sorafenib in HCC by suppressing ferroptosis. Mechanistically, WDR74 was identified as a direct transcriptional target of CAPG. WDR74 disrupted the interaction between p53 and MDM2, resulting in enhanced p53 degradation and increased SLC7A11 expression, thereby suppressing ferroptosis (**Fig. 9M**).

Ferroptosis, a newly recognized form of regulated cell death characterized by excessive lipid peroxidation, has emerged as a critical mechanism of tumor suppression 34. This pathway is particularly relevant in liver diseases, including HCC, owing to the liver's susceptibility to oxidative stress. Although sorafenib, a multikinase inhibitor, is the first-line systemic therapy for advanced HCC, its clinical efficacy is hampered by the development of drug resistance 17. As a well-established ferroptosis inducer, sorafenib has prompted extensive research into its ferroptosis-mediated effects and the discovery of potential therapeutic targets to overcome resistance.

CAPG suppresses ferroptosis in several cancer types, including pancreatic cancer, colorectal cancer, and HCC. Zhao et al. reported that CAPG silencing triggers ferroptosis in colorectal cancer by upregulating the p53 pathway, noting that CAPG knockdown prolongs the half-life of p53, thus stabilizing the protein 35. In pancreatic cancer, Li et al. found that CAPG inhibits the UFMylation of pirin (PIR), thereby downregulating the transcription of GPX4, a key regulator of ferroptosis 36. Zhang et al. further demonstrated that erastin treatment suppresses both mRNA and protein expression of CAPG in HCC, and that ferroptotic stimuli disrupt the binding of HNF4A to the CAPG promoter via KAT2B dissociation 28. In contrast to Zhang et al.'s focus on upstream regulation of CAPG, our study centered on its downstream effects. We revealed that CAPG suppresses ferroptosis through the p53/SLC7A11 axis in HCC, mirroring the mechanism observed in colorectal cancer, rather than via the GPX4 pathway reported in pancreatic cancer. Furthermore, Tsai et al. previously demonstrated that CAPG promotes the malignant behavior of HCC *in vitro* 26. However, the mechanistic details by which CAPG influences HCC proliferation, migration, invasion, and metastasis remain poorly defined, and *in vivo* evidence has been lacking. To our knowledge, no previous studies have examined whether CAPG is involved in sorafenib resistance or whether its oncogenic functions are mediated via ferroptosis. Our findings provide new insight into these unexplored areas and highlight the CAPG-WDR74-p53-SLC7A11 axis as a potential therapeutic target in HCC.

In this study, we integrated proteomics analysis, tissue microarray-based IHC, and public database mining and found that high CAPG expression was associated with poor prognosis in HCC patients. Subsequent *in vitro* and *in vivo* experiments demonstrated that CAPG knockdown inhibited tumor growth and metastasis. Building upon Zhang et al.'s finding that erastin alters CAPG expression, we hypothesized that CAPG may regulate ferroptosis to promote HCC progression. To test this, we measured ferroptosis markers in nude mouse subcutaneous xenografts. CAPG downregulation led to decreased levels of SLC7A11 and GSH, along with increased levels of 4-HNE, lipid peroxidation, and MDA, indicating enhanced ferroptosis. Through lentivirus-mediated CAPG knockdown and overexpression in HCC cell lines, we confirmed that CAPG suppresses sorafenib-induced ferroptosis. Based on this observation, we proposed that CAPG may contribute to sorafenib resistance, which was e validated through *in vitro* data. Furthermore, CAPG expression was significantly upregulated after 24 hours of sorafenib exposure and was elevated in SR HCC cells. Tissue microarray analysis further revealed that patients with HCC who responded to sorafenib treatment exhibited lower CAPG expression levels.

Given that CAPG expression was upregulated following treatment with both sorafenib and erastin, and that previous studies have identified SLC7A11 as a common target of both drugs 37, 38, we hypothesized that CAPG may regulate ferroptosis through SLC7A11. Consistent with prior findings 38, 39, we also observed elevated SLC7A11 expression in SR HCC cells. To investigate the mechanism by which CAPG regulates SLC7A11, we identified 54 potential CAPG-modulating genes via RNA-seq and ChIP-seq analyses. Nrf2, p53, and HIF-1α are common transcriptional regulators of SLC7A11 38, 40, 41. Western blotting revealed a correlation between CAPG and p53 expression but not with NRF2 or HIF-1α. Among the 54 candidate genes, WDR74 was of particular interest. WDR74 has been reported to be upregulated in various cancers, including HCC 42, colorectal cancer 43, lung cancer 44, and melanoma 32, suggesting its role as a tumor-promoting factor. In HCC specifically, Gao et al. demonstrated that WDR74 knockdown suppressed cell proliferation by inhibiting the cell cycle and promoting apoptosis. However, its precise mechanism in HCC remains unclear, and no studies to date have explored its role in ferroptosis 42. Importantly, Li et al. reported that in melanoma, WDR74 promotes tumor progression by modulating RPL5 levels, thereby regulating MDM2 and preventing the ubiquitination and degradation of p53. Based on these findings, we hypothesized that WDR74 may exert a similar function in HCC. We selected WDR74 as a downstream target of CAPG and used dual-luciferase reporter assays and ChIP-qPCR to confirm CAPG binding to the WDR74 promoter. Subsequently, co-IP, p53 ubiquitination, and rescue experiments confirmed that WDR74 inhibits ferroptosis via the p53/SLC7A11 axis by promoting p53 degradation, thereby contributing to HCC progression and sorafenib resistance. In addition, RNA-seq in WDR74-knockdown PLC cells revealed that SLC7A11 was among the overlapping genes when comparing (i) DEGs from CAPG overexpression vs. control, (ii) DEGs from WDR74 knockdown vs. control, and (iii) ferroptosis-related genes. This overlap further supports our findings. Finally, we validated these molecular mechanisms *in vivo* using subcutaneous xenograft models in nude mice derived from 97H-SR cells.

This study has several limitations. First, long-term passaging may cause HCC cell lines to lose key characteristics of the primary tumor. Second, we did not develop or test novel CAPG-targeted therapeutics for *in vivo* validation. Third, the potential influence of CAPG on the HCC immune microenvironment remains uninvestigated. Finally, future multi-center studies with larger patient cohorts are necessary to confirm these findings and facilitate their clinical translation.

In summary, we elucidated a specific molecular mechanism through which CAPG promotes tumor proliferation and sorafenib resistance in HCC. Using both *in vivo* and *in vitro* models, we demonstrated that the CAPG/WDR74/p53/SLC7A11 pathway facilitates HCC progression by suppressing ferroptosis. These findings provide novel insight into the oncogenic role of CAPG and highlight its potential as an effective therapeutic target in HCC.

## Supplementary Material

Supplementary figures and tables.

## Abbreviations

HCC: Hepatocellular carcinoma
TACE: Transcatheter arterial chemoembolization
HAIC: hepatic artery infusion chemotherapy
GPX4: glutathione peroxidase 4
SLC7A11: solute carrier family 7 member 11
GSH: glutathione
CAPG: macrophage-capping protein
bHLH: basic helix-loop-helix
STC-1: stanniocalcin
WDR74: WD repeat-containing protein 74
TP53: tumor protein p53
MDM2: mousedouble minute 2
IHC: immunohistochemistry
OS: overall survival
DEPs: differentially expressed proteins
DMEM: dulbecco's modified Eagle medium
FBS: fetal bovine serum
SR: sorafenib resistant
shRNA: small hairpin RNA
siRNA: small interfering RNA
qRT‒PCR: quantitative real-time polymerase chain reaction
cDNA: Complementary DNA
PMSF: phenylmethylsulfonyl fluorid
ACSL4: acyl-CoA synthetase long-chain family member 4
TFRC: transferrin receptor protein 1
Nrf2: nuclear factor erythroid 2-related factor 2
GAPDH: glyceraldehyde-3-phosphate dehydrogenase
HIF-1α: hypoxia inducible factor 1 alpha
H&E: hematoxylin and eosin
4-HNE: 4-hydroxynonenal
Ab: absorbance
IC50: half-maximal inhibitory concentration
TEM: transmission electron microscopy
OsO_4_: osmium tetroxide
MDA: malondialdehyde
RNA-seq: RNA sequencing
DEGs: differentially expressed genes
ChIP-seq: chromatin immunoprecipitation-sequencing
HEK-293T: human embryonic kidney 293T
co-IP: coimmunoprecipitation
TCGA: the Cancer Genome Atlas
GEO: Gene Expression Omnibus
SD: standard deviation
PFS: progression free survival
Fer-1: ferrostatin-1
IQR: interquartile range
HbsAg: hepatitis B surface antigen
PLT: Platelet
AFP: alpha fetoprotein
CEA: carcinoembryonic antigen
CA199: Carbohydrate antigen199
ALT: alanine aminotransferase
AST: aspartate aminotransferase
γ—GT: gamma-glutamyltranspeptidase
AKP: alkaline phosphatase
