# Supplementary material for: Increased CAPG inhibits ferroptosis to drive tumor proliferation and sorafenib resistance in hepatocellular carcinoma via the WDR74-p53-SLC7A11 pathway
Source: Int J Biol Sci. 2025 Aug 22;21(12):5476–95. doi: 10.7150/ijbs.111419 (PMC12435477; doi:10.7150/ijbs.111419)
Supplement: Supplementary file 1 — Supplementary figures and tables. [file ijbsv21p5476s1.pdf]

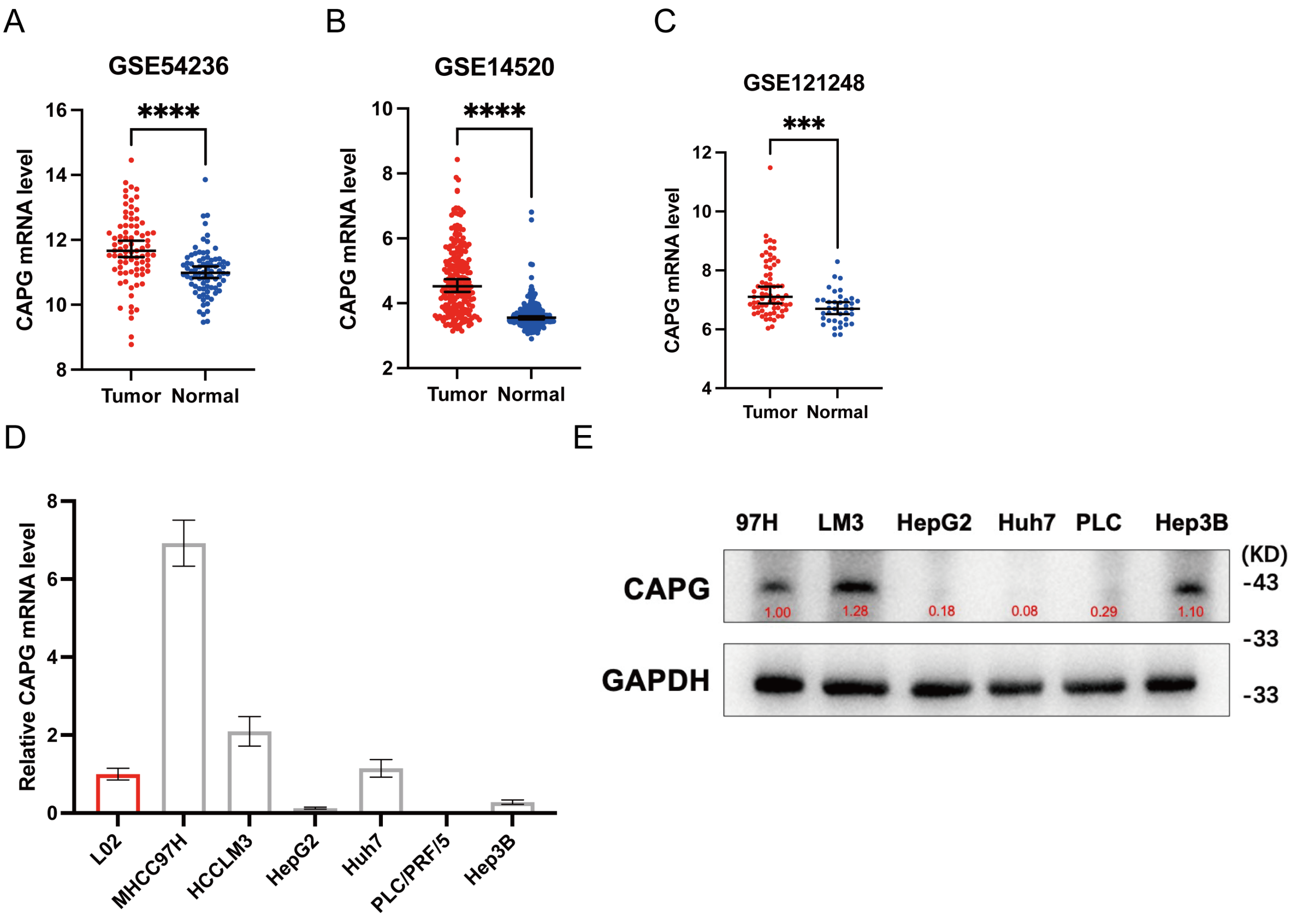

Fig. S1 CAPG is frequently upregulated in HCC.

A-C. Higher CAPG expression level was found in HCC than peritumor tissues based on GSE54236, GSE14520 and GSE121248 database. **D, E.** Relative mRNA and protein expression of CAPG in L02 and multiple HCC cell lines. *P < 0.05, **P < 0.01, ***P < 0.001, ns. no significant.

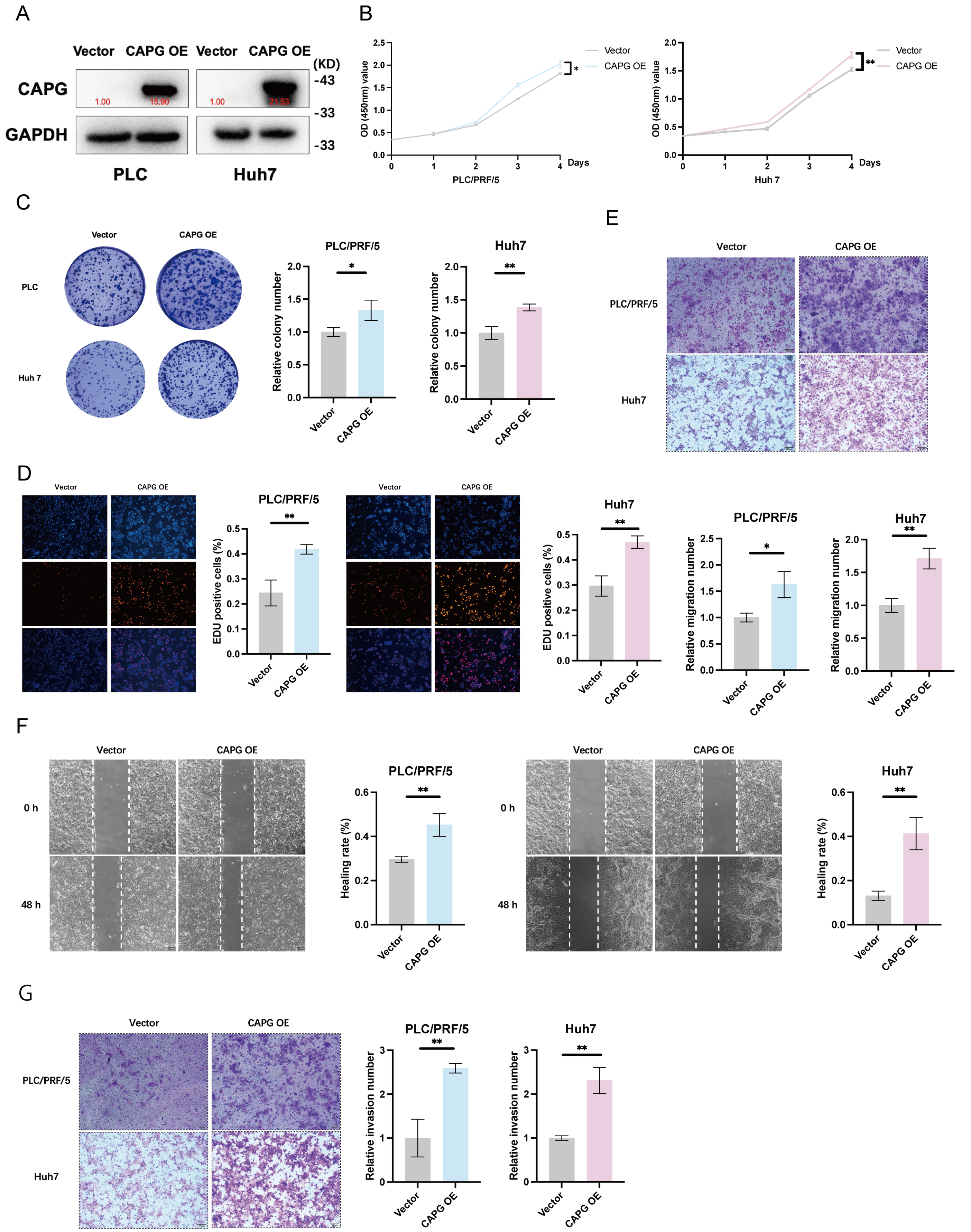

Fig. S2 CAPG enhances HCC cell proliferation, migration and invasion in vitro.
A. Verification the overexpression efficiency of CAPG in HCC cells. **B.** CCK-8 proliferation assays were performed to examine the proliferation rate in CAPG overexpression cells. **C.** Colony formation assays in HCC cells after overexpression of CAPG. **D.** EDU assays in HCC cells after overexpression of CAPG. **E.** The migration rates were determined by transwell migration assays in CAPG overexpression cells. **F.** The migration rates were determined by wound healing assays in

CAPG overexpression cells. **G.** The invasion rates were estimated by matrigel invasion assays in CAPG overexpression cells. *P < 0.05, **P < 0.01, ***P < 0.001, ns. no significant.

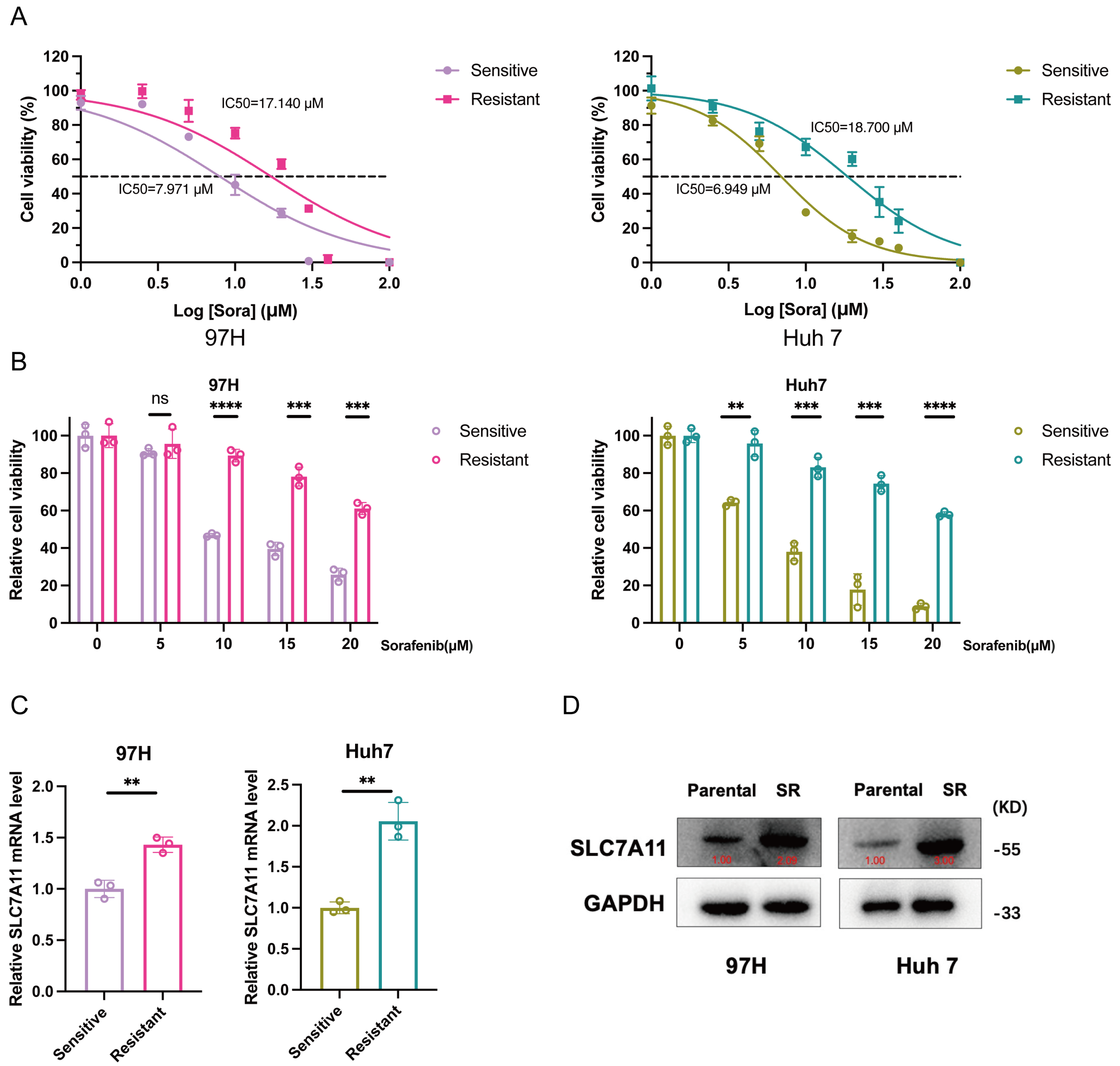

Fig. S3 The characteristics of the sorafenib-resistant HCC cells.

A. The IC50 of 97H, 97HSR, Huh7, and Huh7SR cells to sorafenib. **B.** The CCK-8 assay of relative cell viability of 97H, 97HSR, Huh7, and Huh7SR cells treated with sorafenib at various concentrations for 24 h. **C, D.** Relative mRNA and protein expression of SLC7A11 in 97H, 97HSR, Huh7, and Huh7SR cells. *P < 0.05, **P < 0.01, ***P < 0.001, ns. no significant.

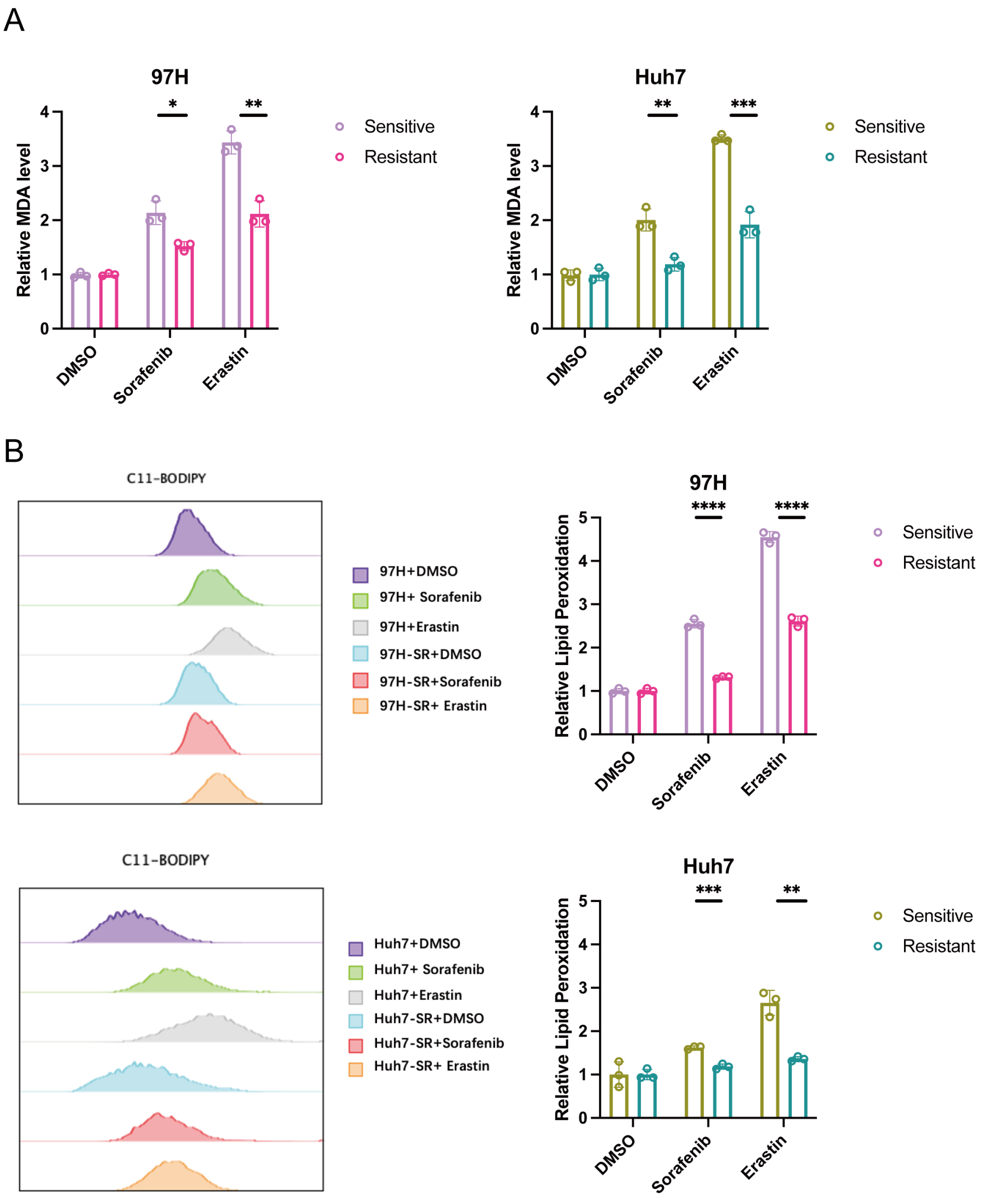

Fig. S4 Sorafenib induces ferroptosis in HCC cells.

A. Relative MDA level of 97H, 97HSR, Huh7, and Huh7SR cells exposed to 10 μM sorafenib and 10 μM erastin. **B.** Lipid ROS levels of 97H, 97HSR, Huh7, and Huh7SR cells exposed to 10 μM sorafenib and 10 μM erastin. *P < 0.05, **P < 0.01, ***P < 0.001, ns. no significant.

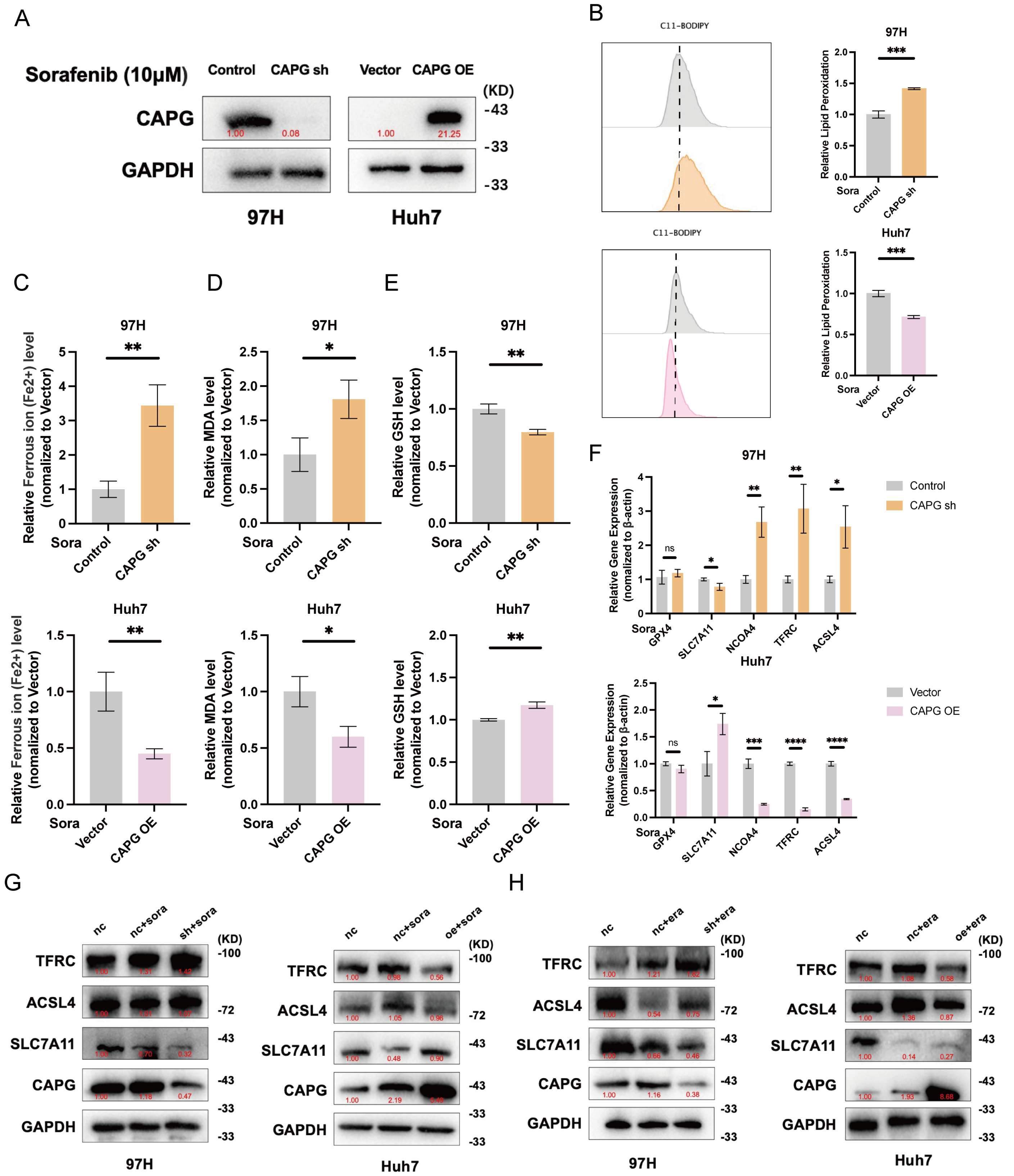

Fig. S5 CAPG overexpression inhibits sorafenib-induced ferroptosis in HCC cells.

A. Silencing and overexpression of CAPG were confirmed by Western blot analyses. **B-E.** Relative lipid ROS levels (**B**), intracellular Fe^{2+} level (**C**), MDA level (**D**) and GSH level (**E**) of CAPG sh and CAPG OE cells exposed to 10 μM sorafenib for 24 h. **F.** Relative ferroptosis related genes mRNA levels of CAPG sh and CAPG OE cells exposed to 10 μM sorafenib for 24 h. **G, H.** Relative ferroptosis related genes protein levels of CAPG sh and CAPG OE cells exposed to 10 μM sorafenib and 10 μM erastin for 24 h. *P < 0.05, **P < 0.01, ***P < 0.001, ns. no significant.

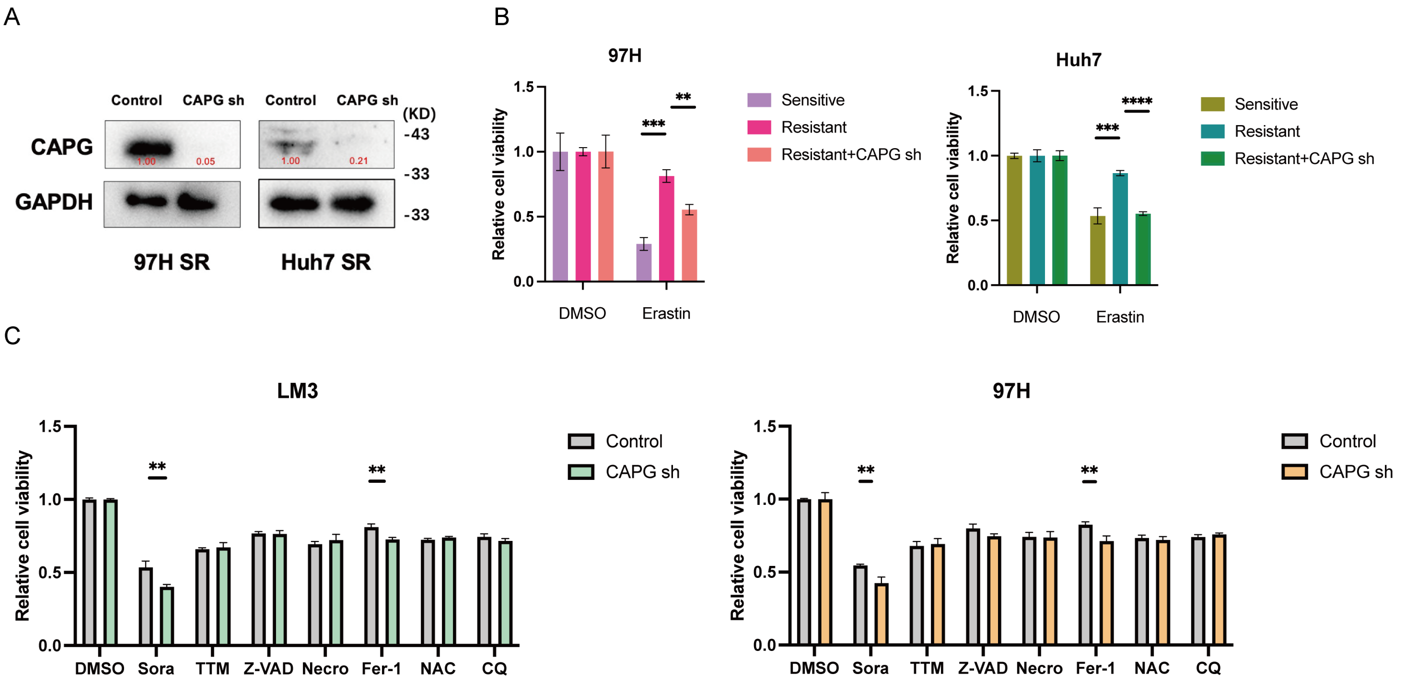

Fig. S6 CAPG knockdown conferred the sensitivity to sorafenib by promoting ferroptosis in resistant HCC cells.

A. Verification the silencing efficiency of CAPG in sorafenib-resistant HCC cells. **B.** Relative cell viability of CAPG-knockdown sorafenib-resistant HCC cells treated with 10 μM sorafenib and 10 μM erastin for 24 h. **C.** CAPG nc and CAPG sh cells were pretreated overnight with 10 uM DMSO, 20 uM TTM, 30 uM Z-VAD-FMK, 20 uM necrostatin-1, 10 uM ferrostatin-1, 1 mM N-acetylcysteine (NAC), and 10 uM chloroquine, respectively. And then cells were treated with 10 uM sorafenib for 24 h with different disposal modes. *P < 0.05, **P < 0.01, ***P < 0.001, ns. no significant.

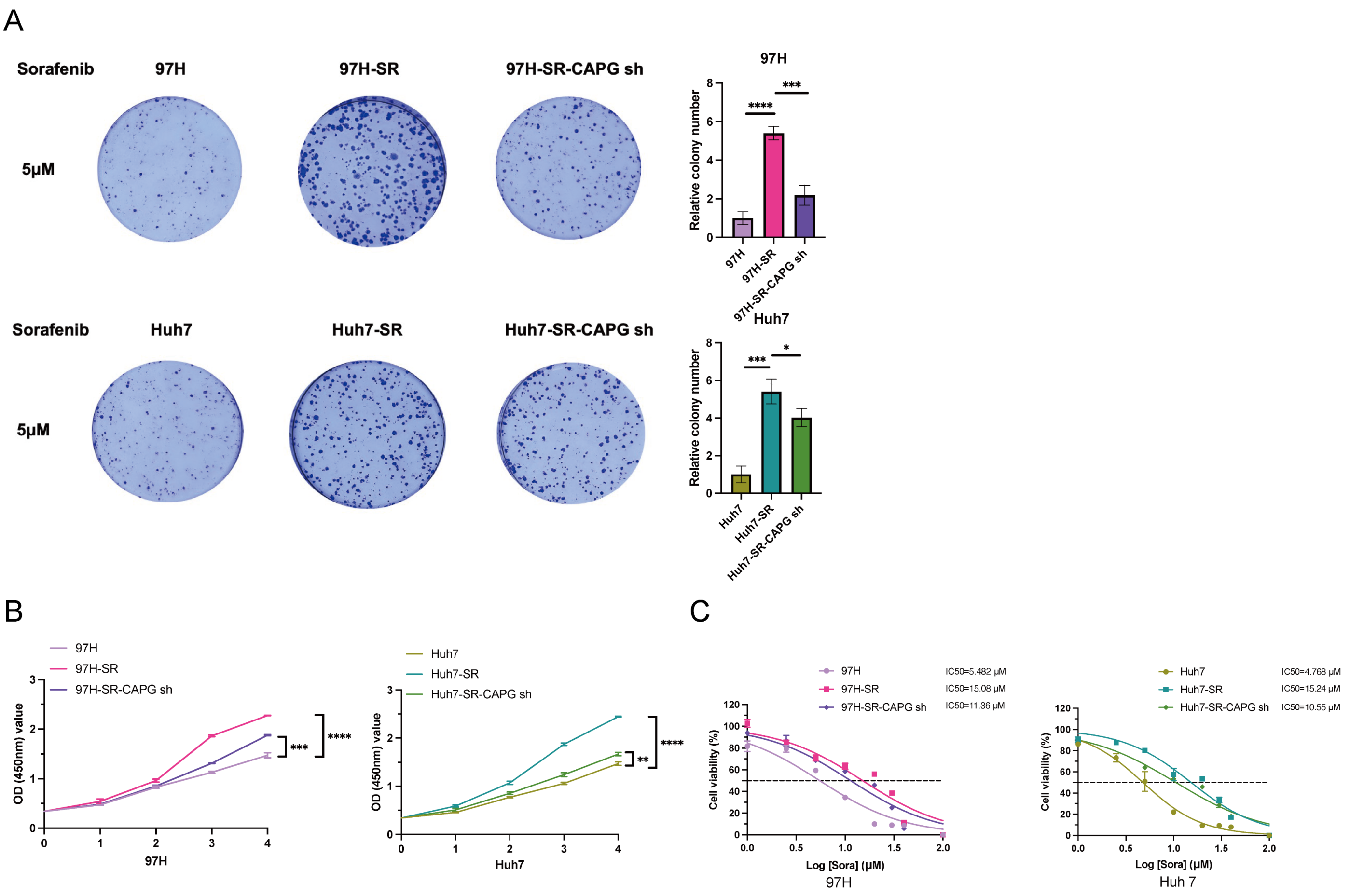

Fig. S7 CAPG knockdown conferred the sensitivity to sorafenib by promoting ferroptosis in resistant HCC cells.

A. Colony formation assay of HCC cells, sorafenib-resistant HCC cells and CAPG-knockdown sorafenib-resistant HCC cells treated with sorafenib (5 μM) for 24 h. **B.** The OD value of CAPG-knockdown sorafenib-resistant HCC cells treated with sorafenib (10 μM) for 24 h. **C.** The IC50 of CAPG-knockdown sorafenib-resistant HCC cells treated with various concentrations of sorafenib for 24 h. *P < 0.05, **P < 0.01, ***P < 0.001, ns. no significant.

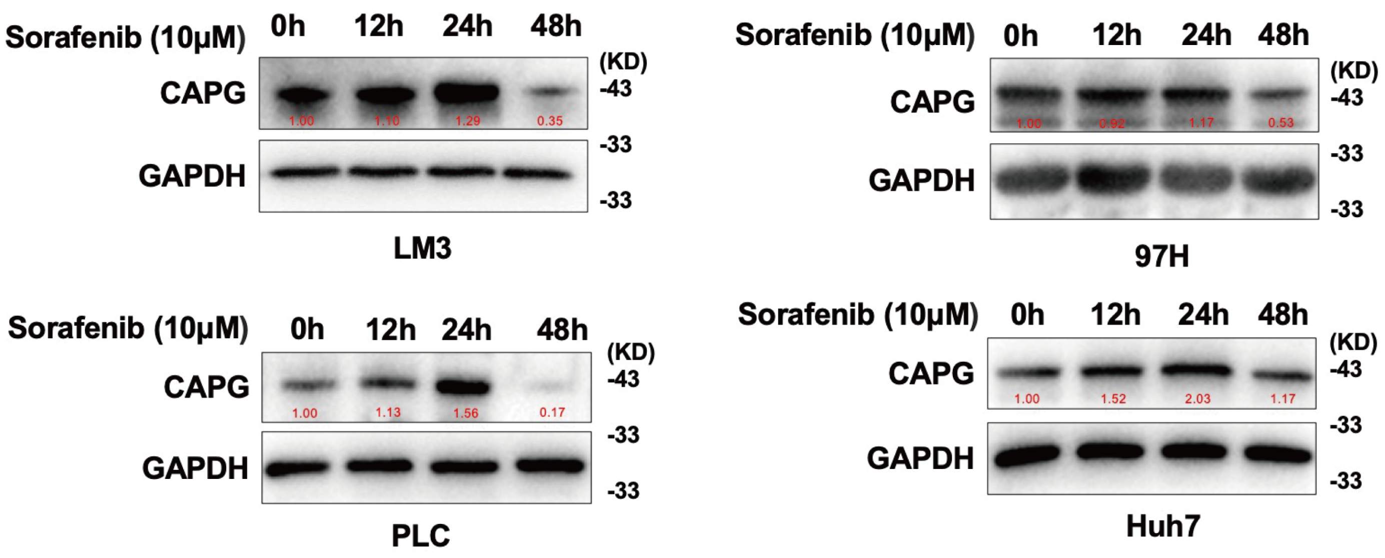

Fig. S8 HCC cells incubated with 10 μM sorafenib for different hours, analyzing CAPG protein levels by western blots.

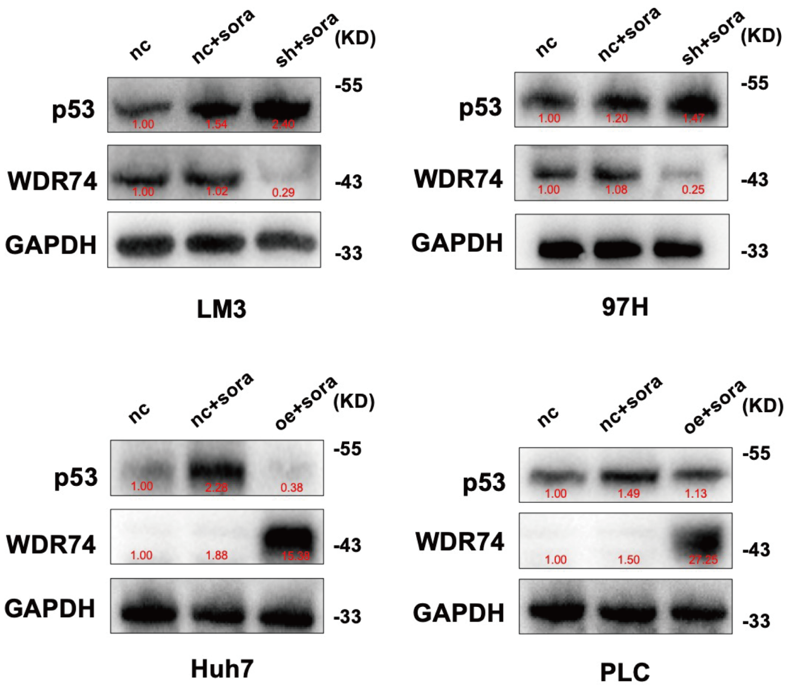

Fig. S9 The negative expression relationship between WDR74 and TP53 in HCC cells.

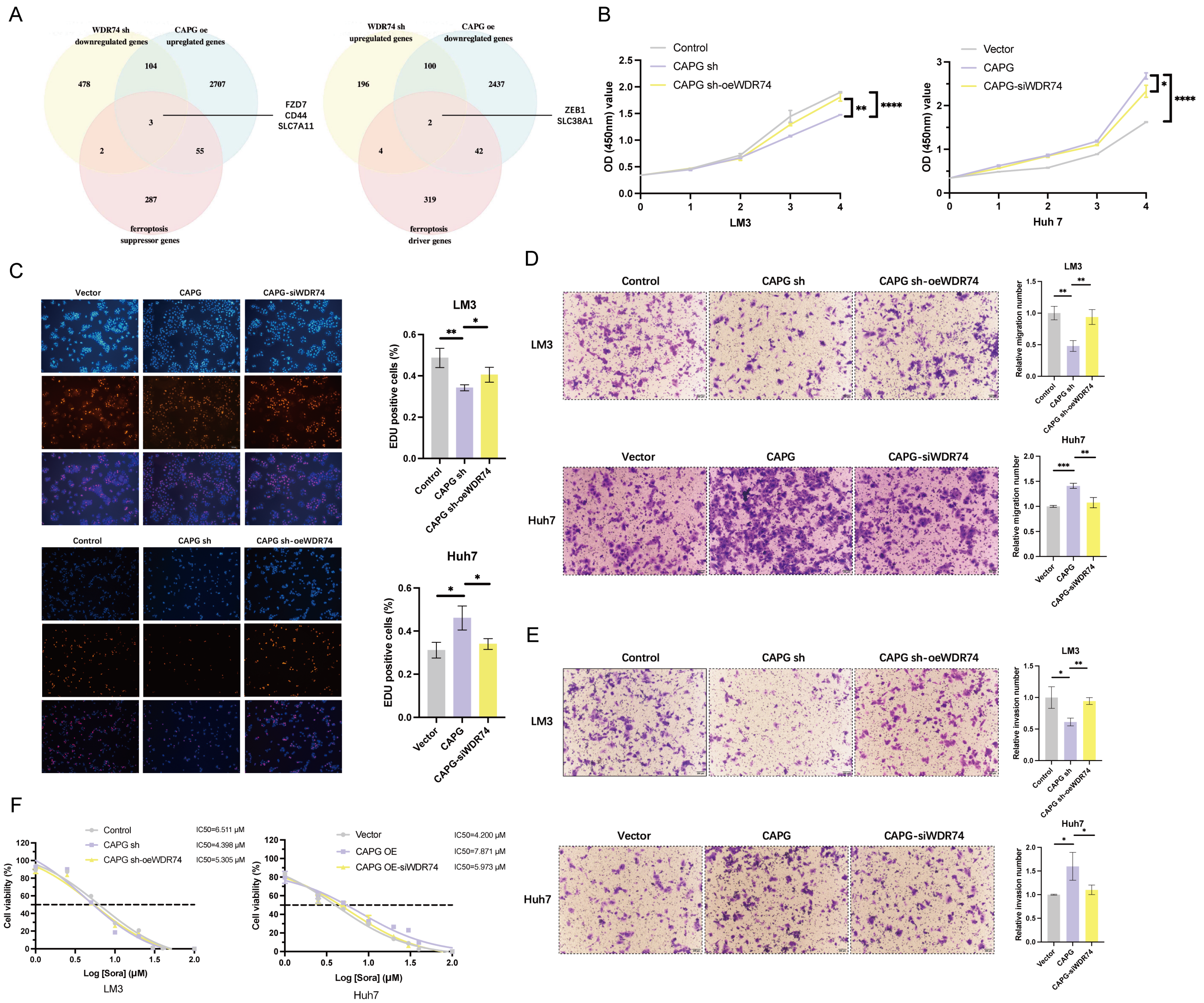

Fig. S10 Knockdown of WDR74 enhances CAPG induced malignant phenotype of HCC.
A. Venn diagram displays the overlap analysis among DEGs in CAPG OE PLC/PRF/5 cells, DEGs in WDR74 sh PLC/PRF/5 cells, and ferroptosis related genes. **B-F.** CCK-8 proliferation assays (**B**), EDU assays (**C**), transwell migration assays (**D**), matrigel invasion assays (**E**) and IC50 (**F**) of CAPG sh cells with or without WDR74 overexpression and CAPG OE cells with or without WDR74 knockdown. *P < 0.05, **P < 0.01, ***P < 0.001, ns. no significant.

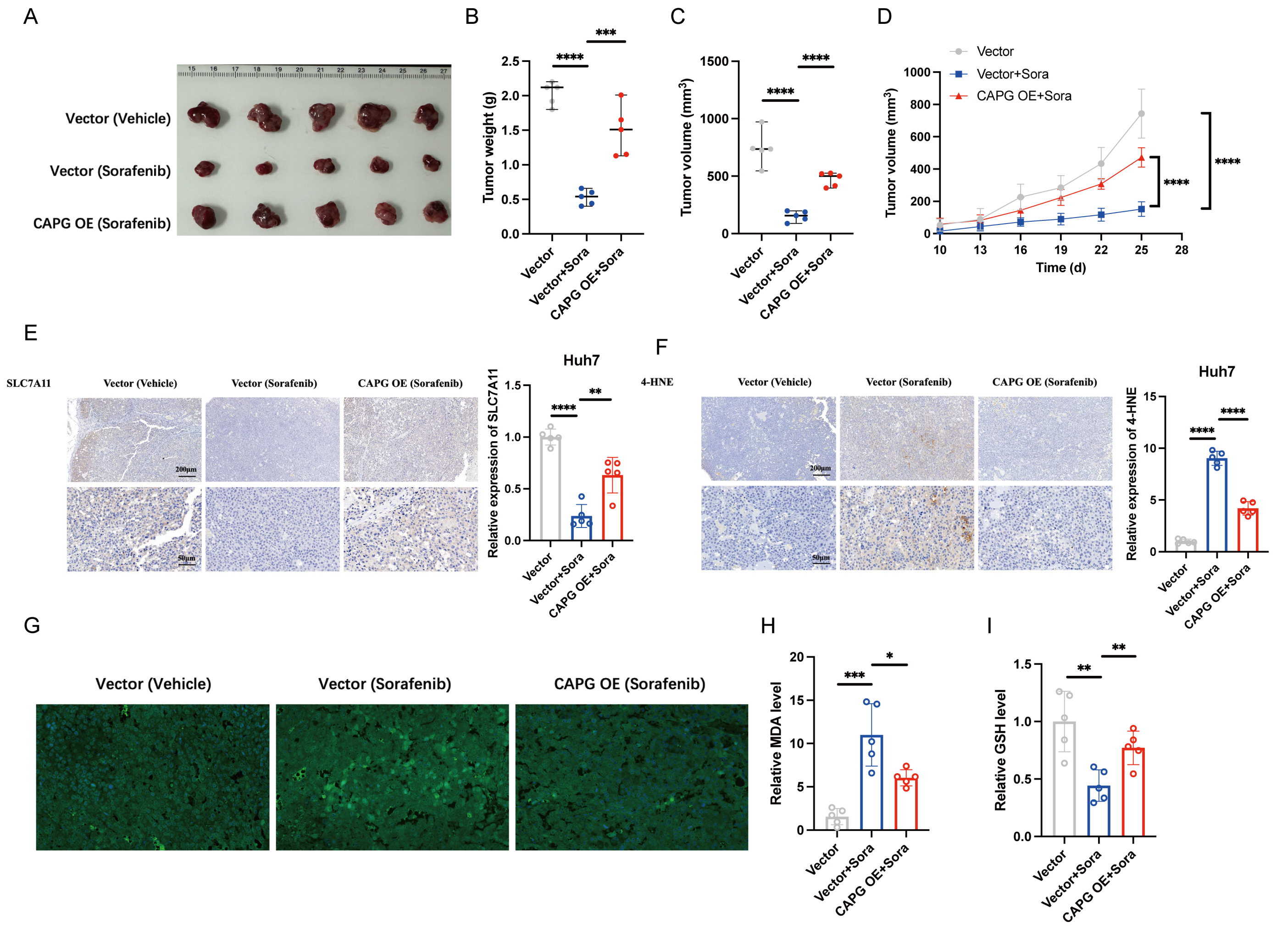

Fig. S11. CAPG overexpression inhibits sorafenib induced ferroptosis in vivo.

A. Representative images of the tumors derived from BALB/c nude mice transplanted with 5×10^6 Huh7 and stable Huh7 OE-CAPG cells with the treatment of saline or sorafenib. **B.** Tumor weight in each group (n =5). **C.** Tumor volume in each group (n =5). **D.** Tumor growth curve (n =5). **E, F.** Representative pictures of IHC staining (SLC7A11 and 4-HNE) for tumors. **G.** Representative IF staining with the C11-BODIPY probe for tumors. **H.** Relative MDA level in each group (n =5). **I.** Relative GSH level in each group (n =5). *P < 0.05, **P < 0.01, ***P < 0.001, ns. no significant.

Table S1. Clinical characteristics of the sorafenib sensitive group and the sorafenib resistant group.

Variables	Number (proportion, %) or median (IQR)		P*
	The sorafenib sensitive group (n = 49)	The sorafenib resistant group (n = 28)	
Age, ≥ 50/< 50 (years)	18/31 (36.7/63.3)	12/16 (42.9/57.1)	0.596
Sex, Male/Female	46/3 (93.9/6.1)	26/2 (92.9/7.1)	0.861
Tumor number, Multiple/Single	5/44 (/10.2/89.8)	0/28 (0.0/100.0)	0.101
Maximum tumor size, cm	7.1 (3.9-10.0)	6.4 (4.4-12.2)	0.531
Tumor thrombus, Positive/Negative	35/14 (71/4/28.6)	22/6 (78.6/21.4)	0.492
TNM stage, I/II/III/IV	11/33/3/2 (22.4/67.3/6.1/4.1)	6/21/0/1 (21.4/75.0/0.0/3.6)	0.608
HbsAg, Positive/Negative	42/7 (85.7/14.3)	22/6 (78.6/21.4)	0.482
PLT, ×10^9/L	167 (126.5-213.0)	205.0 (137.3-275.8)	0.140
AFP, ng/ml	125.7 (31.6-757.7)	768.1 (50.0-1210.0)	0.494
CEA, ng/ml	2.00 (1.35-2.75)	2.45 (1.45-3.88)	0.109
CA199, KU/L	15.5 (7.7-28.6)	15.0 (8.4-22.6)	0.655
Prothrombin time, s	12.4 (11.9-13.5)	12.2 (11.8-13.3)	0.643
Total bilirubin, μmol/L	15.8 (12.1-19.9)	18.8 (12.5-24.4)	0.232
Albumin level, g/L	38.6 (34.4-41.7)	36.8 (34.4-41.2)	0.760
ALT, U/L	58.4 (34.6-98.2)	42.0 (33.8-87.4)	0.185
AST, U/L	37.8 (28.8-52.8)	33.0 (26.1-70.3)	0.309
γ-GT, U/L	78.0 (50.0-177.5)	115.5 (56.3-153.3)	0.670
AKP, U/L	84.0 (71.0-122.5)	98.5 (78.3-118.5)	0.955
CAPG expression, high/low	26/23 (53.1/46.9)	23/5 (82.1/17.9)	0.011
Overall survival, days	505 (309-703)	248 (121-430)	< 0.001
Progression free survival, days	446 (190-579)	81 (53-98)	< 0.001

IQR, interquartile range; HbsAg, hepatitis B surface antigen; PLT, Platelet; AFP, alpha fetoprotein; CEA, carcinoembryonic antigen; CA199, Carbohydrate antigen199; ALT, alanine aminotransferase; AST, aspartate aminotransferase; γ – GT, gamma-glutamyltranspeptidase; AKP, alkaline phosphatase.

Table S2. The shRNA and siRNA sequences.

Name	TargetSeq
sh-CAPG#1	GGCAATGAGTCTGACCTCTTC
sh-CAPG#2	GCATTTCACAAGACCTCCACA
si-WDR74#1	GGUCCGUGUUUAUGAUCCA
sh-p53#1	CGGCGCACAGAGGAAGAGAAT
sh-SLC7A11#1	CCTGTCACTATTTGGAGCTTT

Table S3. Primer sequences used in this study.

Name	Forward sequence, 5' to 3'	Reverse sequence, 3' to 5'
β-actin	CATGTACGTTGCTATCCAGGC	CTCCTTAATGTCACGCACGAT
CAPG	GGGGACTCCTACCTAGTGCTG	CACCACCTTCCTGGTACTTGA
GPX4	GAGGCAAGACCGAAGTAAACTAC	CCGAACTGGTTACACGGGAA
SLC7A11	TCTCCAAAGGAGGTTACCTGC	AGACTCCCCTCAGTAAAGTGAC
NCOA4	GAGGTGTAGTGATGCACGGAG	GACGGCTTATGCAACTGTGAA
TFRC	ACCATTGTCATATACCCGGTTCA	CAATAGCCCAAGTAGCCAATCAT
ACSL4	CATCCCTGGAGCAGATACTCT	TCACTTAGGATTTCCCTGGTCC
WDR74	CCTGGGGTGTGTAGGATGC	CAAGTCCAGCCAGTCATTCCG

Table S4. TMT-labeled quantitative proteomics identified differentially expressed proteins (DEPs) in HCC tissues versus normal controls, defined as proteins with >1.6-fold increase or <0.625-fold decrease (p<0.05).

Accession	Protein Name	Gene Name	average T-T-1	average T-N-1	T-T-1/T-N-1	t test p value
Q96GP6	Scavenger receptor class F member 2	SCARF2	1.21350	0.36831	3.29480	0.02598
P02461	Collagen alpha-1(III) chain	COL3A1	1.57486	0.65757	2.39495	0.03130
P02452	Collagen alpha-1(I) chain	COL1A1	1.65319	0.73143	2.26020	0.03553
Q05707	Collagen alpha-1(XIV) chain	COL14A1	1.26025	0.63246	1.99263	0.02226
P08123	Collagen alpha-2(I) chain	COL1A2	1.54096	0.78871	1.95377	0.04400
P20702	Integrin alpha-X	ITGAX	1.69736	0.88433	1.91938	0.01284
O00258	Guided entry of tail-anchored proteins factor 1	GET1	2.62894	1.40519	1.87088	0.00303
Q96D15	Reticulocalbin-3	RCN3	1.54170	0.84689	1.82043	0.01413
Q96AY3	Peptidyl-prolyl cis-trans isomerase FKBP10	FKBP10	0.32964	0.18394	1.79211	0.04330
Q8TB45	DEP domain-containing mTOR-interacting protein	DEPTOR	1.28029	0.72404	1.76828	0.02393
P40121	Macrophage-capping protein	CAPG	1.07910	0.61862	1.74437	0.00339
Q9H6X2	Anthrax toxin receptor 1	ANTXR1	2.57843	1.50661	1.71141	0.01415
O43148	mRNA cap guanine-N7 methyltransferase	RNMT	0.12918	0.07587	1.70257	0.00929
P24043	Laminin subunit alpha-2	LAMA2	0.14740	0.08683	1.69758	0.02398
P00488	Coagulation factor XIII A chain	F13A1	1.29121	0.76453	1.68889	0.01874
Q9ULR3	Protein phosphatase 1H	PPM1H	2.56470	1.52968	1.67663	0.00653
P50454	Serpin H1	SERPINH1	1.44548	0.87147	1.65868	0.00059
O43520	Phospholipid-transporting ATPase IC	ATP8B1	1.44810	0.87754	1.65018	0.04806
Q16643	Drebrin	DBN1	0.56416	0.34467	1.63681	0.01112
Q8N131	Porimin	TMEM123	0.53751	0.32949	1.63134	0.02292
P54709	Sodium/potassium-transporting ATPase subunit beta-3	ATP1B3	0.97711	0.60491	1.61530	0.00515
P54868	Hydroxymethylglutaryl-CoA synthase, mitochondrial	HMGCS2	0.65919	1.05490	0.62488	0.03599
P03928	ATP synthase protein 8	MT-ATP8	0.69807	1.12279	0.62173	0.00062
Q9Y283	Inversin	INVS	0.62567	1.00668	0.62152	0.00251
P36537	UDP-glucuronosyltransferase 2B10	UGT2B10	0.71414	1.15232	0.61975	0.01400
P35573	Glycogen debranching enzyme	AGL	0.77002	1.24383	0.61907	0.01136
Q16851	UTP--glucose-1-phosphate uridylyltransferase	UGP2	0.69254	1.12076	0.61792	0.00087
Q9NYL5	24-hydroxycholesterol 7-alpha-hydroxylase	CYP39A1	0.40404	0.65552	0.61637	0.01248
Q9P2D8	Protein unc-79 homolog	UNC79	1.54580	2.51359	0.61498	0.01843
Q9Y2D2	UDP-N-acetylglucosamine transporter	SLC35A3	0.65076	1.06206	0.61273	0.00579

Q9UBX1	Cathepsin F	CTSF	1.16258	1.90442	0.61046	0.03263
P47989	Xanthine dehydrogenase/oxidase	XDH	0.58107	0.95224	0.61021	0.00414
Q9H227	Cytosolic beta-glucosidase	GBA3	0.77537	1.27802	0.60670	0.02502
P23378	Glycine dehydrogenase (decarboxylating), mitochondrial	GLDC	1.03992	1.71523	0.60628	0.04468
Q3LXA3	Triokinase/FMN cyclase	TKFC	0.75613	1.25009	0.60486	0.00738
Q9NRI5	Disrupted in schizophrenia 1 protein	DISC1	1.55967	2.59113	0.60193	0.00266
O43708	Maleylacetoacetate isomerase	GSTZ1	0.64574	1.07360	0.60147	0.00551
Q9UJT0	Tubulin epsilon chain	TUBE1	0.26854	0.44752	0.60007	0.00099
Q9NPD5	Solute carrier organic anion transporter family member 1B3	SLCO1B3	1.05058	1.75396	0.59897	0.00649
Q3V5L5	Alpha-1,6-mannosylglycoprotein 6-beta-N-acetylglucosaminyltransferase B	MGAT5B	0.64522	1.08088	0.59694	0.00473
O96033	Molybdopterin synthase sulfur carrier subunit	MOCS2	1.17070	1.96292	0.59641	0.00053
P16662	UDP-glucuronosyltransferase 2B7	UGT2B7	0.61816	1.03945	0.59470	0.00536
P08263	Glutathione S-transferase A1	GSTA1	1.22217	2.06022	0.59322	0.01021
Q9NWW7	Uncharacterized protein C2orf42	C2orf42	1.53166	2.58204	0.59320	0.01776
P06737	Glycogen phosphorylase, liver form	PYGL	0.68184	1.15317	0.59128	0.00636
P05062	Fructose-bisphosphate aldolase B	ALDOB	0.76497	1.29467	0.59086	0.04080
O00757	Fructose-1,6-bisphosphatase isozyme 2	FBP2	0.68923	1.16860	0.58979	0.00630
P08319	All-trans-retinol dehydrogenase [NAD(+)] ADH4	ADH4	0.81654	1.38486	0.58962	0.01204
P07327	Alcohol dehydrogenase 1A	ADH1A	0.68681	1.16575	0.58916	0.00105
O75452	Retinol dehydrogenase 16	RDH16	0.69314	1.17815	0.58834	0.00354
Q96DG6	Carboxymethylenebutenolidase homolog	CMBL	0.70834	1.21562	0.58270	0.01154
P09210	Glutathione S-transferase A2	GSTA2	0.66354	1.14114	0.58146	0.01266
Q9Y6L6	Solute carrier organic anion transporter family member 1B1	SLCO1B1	0.52092	0.89660	0.58099	0.00017
P54840	Glycogen [starch] synthase, liver	GYS2	0.71192	1.22728	0.58008	0.03266
O14756	17-beta-hydroxysteroid dehydrogenase type 6	HSD17B6	0.69612	1.20183	0.57922	0.00422
O94788	Retinal dehydrogenase 2	ALDH1A2	0.75123	1.30045	0.57767	0.02050
Q93088	Betaine--homocysteine S-methyltransferase 1	BHMT	0.72507	1.27188	0.57008	0.00970

Q9H2A2	2-aminomuconic semialdehyde dehydrogenase	ALDH8A1	0.81605	1.43892	0.56713	0.00081
Q9NYQ3	Hydroxyacid oxidase 2	HAO2	0.26694	0.47507	0.56189	0.01858
P30837	Aldehyde dehydrogenase X, mitochondrial	ALDH1B1	0.64122	1.14265	0.56117	0.01898
Q15800	Methylsterol monooxygenase 1	MSMO1	0.71236	1.27109	0.56043	0.04258
P51857	Aldo-keto reductase family 1 member D1	AKR1D1	0.74875	1.34278	0.55761	0.00221
Q86YJ6	Threonine synthase-like 2	THNSL2	0.82016	1.48704	0.55154	0.00816
Q96N76	Urocanate hydratase	UROC1	0.69751	1.27546	0.54687	0.00036
O95718	Steroid hormone receptor ERR2	ESRRB	0.48937	0.89918	0.54424	0.01583
Q96HN2	Adenosylhomocysteinase 3	AHCYL2	1.43582	2.68885	0.53399	0.00038
O95954	Formimidoyltransferase-cyclodeaminase	FTCD	0.67900	1.29577	0.52401	0.02224
Q9Y6V0	Protein piccolo	PCLO	0.78505	1.53386	0.51182	0.01776
Q9BZV2	Thiamine transporter 2	SLC19A3	0.41400	1.00245	0.41299	0.00346
